# Remodeling of Lipid A in *Pseudomonas syringae* pv. *phaseolicola* In Vitro

**DOI:** 10.3390/ijms23041996

**Published:** 2022-02-11

**Authors:** Tim Gerster, Michelle Wröbel, Casey E. Hofstaedter, Dominik Schwudke, Robert K. Ernst, Stefanie Ranf, Nicolas Gisch

**Affiliations:** 1 Chair of Phytopathology, TUM School of Life Sciences Weihenstephan, Technical University of Munich, 85354 Freising-Weihenstephan, Germany; tim.gerster@tum.de; 2 Division of Bioanalytical Chemistry, Priority Area Infections, Research Center Borstel, Leibniz Lung Center, 23845 Borstel, Germany; mwroebel@fz-borstel.de (M.W.); dschwudke@fz-borstel.de (D.S.); 3 Department of Microbial Pathogenesis, School of Dentistry, University of Maryland, Baltimore, MD 21201, USA; casey.hofstaedter@som.umaryland.edu (C.E.H.); rkernst@umaryland.edu (R.K.E.); 4 German Center for Infection Research (DZIF), Thematic Translational Unit Tuberculosis, Partner Site Hamburg-Lübeck-Borstel-Riems, 23845 Borstel, Germany; 5 Airway Research Center North, Member of the German Center for Lung Research (DZL), Site Research Center Borstel, 23845 Borstel, Germany

**Keywords:** lipid A, lipopolysaccharide, Pseudomonas, lipopolysaccharide remodeling, mass spectrometry

## Abstract

*Pseudomonas* species infect a variety of organisms, including mammals and plants. Mammalian pathogens of the Pseudomonas family modify their lipid A during host entry to evade immune responses and to create an effective barrier against different environments, for example by removal of primary acyl chains, addition of phosphoethanolamine (*P*-EtN) to primary phosphates, and hydroxylation of secondary acyl chains. For *Pseudomonas syringae* pv. *phaseolicola* (*Pph*) 1448A, an economically important pathogen of beans, we observed similar lipid A modifications by mass spectrometric analysis. Therefore, we investigated predicted proteomes of various plant-associated *Pseudomonas* spp. for putative lipid A-modifying proteins using the well-studied mammalian pathogen *Pseudomonas aeruginosa* as a reference. We generated isogenic mutant strains of candidate genes and analyzed their lipid A. We show that the function of PagL, LpxO, and EptA is generally conserved in *Pph* 1448A. PagL-mediated de-acylation occurs at the distal glucosamine, whereas LpxO hydroxylates the secondary acyl chain on the distal glucosamine. The addition of *P*-EtN catalyzed by EptA occurs at both phosphates of lipid A. Our study characterizes lipid A modifications in vitro and provides a useful set of mutant strains relevant for further functional studies on lipid A modifications in *Pph* 1448A.

## 1. Introduction

The Gram-negative plant pathogen *Pseudomonas syringae* infects a wide range of economically important crop species. Severe, worldwide disease outbreaks caused by *P. syringae* have long prompted researchers to study this pathogen. As a result, *P. syringae* is one of the best-studied plant pathogens for investigating molecular mechanisms of pathogenicity and pathogen–host interactions [1]. *P. syringae* pv. *phaseolicola* (*Pph*) causes a halo blight of common beans, which leads to major yield losses in China, Australia and developing countries [2,3,4].

A major characteristic of Gram-negative bacteria is the presence of lipopolysaccharide (LPS) in the cell envelope. The LPS structure can be divided into three parts: the O-polysaccharide (OPS), a core oligosaccharide, and the lipid A, which anchors the molecule in the outer leaflet of the outer cell membrane. While the OPS and the core oligosaccharide shield the bacterium from the environment and protect it from hydrophobic molecules, the hydrophobic lipid A inhibits the passage of hydrophilic molecules. The interplay of all three parts of the LPS provides Gram-negative bacteria with an effective barrier against a variety of harmful compounds [5,6,7,8].

Although lipid A biosynthesis is conserved among Gram-negative bacteria, its structure varies amongst different species [9,10]. Lipid A is synthesized on the cytoplasmic side of the inner membrane. The sugar molecule uridine diphosphate *N*-acetylglucosamine (UDP-D-GlcNAc) is the initial molecule for lipid A biosynthesis. As a first step, LpxA catalyzes the addition of a primary acyl chain to the 3-OH group of UDP-D-GlcNAc (brief summary of the biosynthesis is depicted in Figure S1). Deacetylation by LpxC provides the precursor for the addition of a second acyl chain to the free amine substrate catalyzed by LpxD, thus resulting in UDP-2,3-diacyl-GlcN. Removal of a uridine monophosphate from the UDP-2,3-diacyl-GlcN by LpxH is necessary to form a mono-phosphorylated GlcN (also named lipid X). Condensation of lipid X and UDP-2,3-diacyl-GlcN catalyzed by LpxB forms a tetraacyl-β-(1→6)-di-GlcN monophosphate. Phosphorylation at the 4′ position by LpxK results in lipid IV_A_. Essential for the progression of the lipid A synthesis is the addition of the 3-deoxy-D-*manno*-oct-2-ulosonic acid (Kdo) moieties [9,11]. The lipid A biosynthesis is completed by the addition of secondary acyl chains catalyzed by LpxL and LpxM [12,13]. In *Pseudomonas* spp., the lipid A comprises a di-phosphorylated β-(1→6)-linked di-glucosamine backbone. Two amide-bound 3-hydroxydodecanoic (3-OH-C12:0) and two ester-bound 3-hydroxydecanoic acids (3-OH-C10:0) are linked to the backbone as primary acyl chains, respectively. As secondary fatty acids 2-hydroxydodecanoic (2-OH-C12:0) or dodecanoic (C12:0; lauric) acids are O-linked to the two primary 3-OH-C12:0 acids [14,15]. In *Escherichia coli* and *Salmonella* lipid A, 3-hydroxytetradecanoic (3-OH-C14:0) and 3-OH-C12:0 are attached as primary and secondary acyl chains, respectively, to the di-phosphorylated β-(1→6)-linked di-glucosamine backbone [16].

Upon synthesis, the basic hexa-acylated lipid A can be modified in various ways by bacterial enzymes. This remodeling process enables Gram-negative bacteria to adapt to changes in environmental conditions, such as pH, ion concentrations, or the presence of cationic antimicrobial peptides (CAMPs), and promote bacterial virulence [17]. *Pseudomonas* spp. modify their lipid A, for example, by adding 4-amino-4-deoxy-L-arabinose (L-Ara4N) and phosphoethanolamine (*P*-EtN) to primary phosphates or a hexadecanoic acid (C16:0; palmitic acid) as an additional acyl chain, as well as by hydroxylation of secondary and/or de-acylation of primary ester-linked acyl chains [14,18,19,20]. Modifying negatively charged phosphates at the 1 and 4′ position of the di-glucosamine backbone with positively charged L-Ara4N or *P*-EtN substituents neutralizes their charge. Neutral charges confer resistance against CAMPs that use negatively charged phosphates for initial electrostatic interactions [21]. Transfer of L-Ara4N and *P*-EtN to lipid A is catalyzed by ArnT (PmrK) and EptA, respectively, which are regulated by the PmrA/PmrB (BasS/BasR) two-component system (TCS) in *Salmonella* spp. and *E*. *coli* [22,23]. *P*-EtN transferases utilize phosphatidylethanolamine as donor and transfer *P*-EtN to multiple positions of surface structures of Gram-negative bacteria. Such *P*-EtN acceptors are lipid A and the core region of LPS, as well as bacterial surface proteins such as flagella and pilin structures [24,25,26,27,28,29]. EptA mediated *P*-EtN transfer occurs at the periplasmic side of the inner membrane. In *Salmonella* and *E*. *coli*, the 1-phosphate of lipid A is predominantly modified [30,31]. In *P. aeruginosa* PA14, EptA only modifies the 4′-phosphate and this process is regulated by the ColR/ColS TCS [32]. *P*-EtN additions not only confer resistance against CAMPs but are also critical for maintaining the integrity of the outer membrane. In *Citrobacter rodentium* loss of *eptA* leads to an enhanced formation of outer membrane vesicles (OMV) compared to the wild type (WT) [33]. The undecaprenyl-phosphate-linked L-Ara4N is synthesized in the cytoplasm by Ugd, ArnA, ArnB, ArnC, and ArnD and transported across the inner membrane by ArnE and ArnF. The transfer of L-Ara4N to lipid A is then catalyzed by ArnT on the periplasmic side of the inner membrane [11].

PagP is an acyltransferase initially identified in *Salmonella* and later in other bacteria including *P. aeruginosa* and *E. coli* [19,34,35]. It is located in the outer membrane where it catalyzes the palmitoylation of lipid A. Palmitate is transferred from the *sn*-1 position of phospholipids to the N-linked (*R*)-3-hydroxymyristate chain on the proximal glucosamine unit of lipid A in *Salmonella* [35]. PagL is located in the outer membrane as well and hydrolyzes an ester-linked acyl chain at the *O*-3 position of the distal glucosamine of lipid A in *P. aeruginosa* PAO1 [18,36]. Removal of an acyl chain increases membrane fluidity and confers resistance to CAMPs [37]. In *Salmonella enterica,* constitutive PagL expression enhances outer membrane vesicle formation, resulting in overvesiculation, whereas a *pagL* deletion strain exhibits decreased OMV secretion [38]. The dioxygenase LpxO, located at the inner membrane, catalyzes the hydroxylation of secondary acyl chains in *P. aeruginosa* PAO1. This leads to increased resistance to CAMPs and is required for full virulence. Hydroxylation-proficient bacteria usually carry one copy of *lpxO* in their genome [11,39]; however, laboratory-adapted and clinical isolates may have two copies of *lpxO*. These two hydroxylation events can be attributed to two different *lpxO* orthologs (*lpxO1* and *lpxO2*); however, their functionality has not been assigned to a specific position [14].

In *Salmonella*, the PhoP/PhoQ TCS regulates PagP, PagL, and LpxO, resulting in addition, removal, and hydroxylation of acyl chains of the lipid A moiety, respectively [17]. The TCSs PhoP/PhoQ and PmrA/PmrB respond to environmental stimuli such as Mg^2+^, Ca^2+^, Fe^3+^, CAMPs, and pH changes. PhoQ, a transmembrane histidine kinase, is autophosphorylated upon sensing physiological signals and subsequently phosphorylates the corresponding response regulator PhoP [40,41,42,43,44]. The PhoP/PhoQ TCS is important for *Xylella fastidiosa* survival in planta as well as biofilm formation and cell–cell aggregation [45]. PmrB is another sensor kinase responding to environmental stimuli, and PmrA is the corresponding response regulator. In *Salmonella*, the PmrA/PmrB TCS is also activated by PhoP/PhoQ. PmrA/PmrB regulates the expression of ArnT and *P*-EtN transferases in *Salmonella* and *E. coli*, but not in *P. aeruginosa* [17,32,46].

While lipid A biosynthesis and modifications have been studied in detail in many animal and human pathogens, these pathways are still largely unknown in plant-associated bacteria, including economically important plant pathogens of the *P. syringae* species complex. Here, we investigated lipid A structure modifications and the underlying lipid A-modifying enzymes as well as their regulators in the bean pathogen *Pph* 1448A. We identified the orthologous remodeling genes *pagL*, *lpxO*, and *eptA* of *P. aeruginosa* PAO1 in *Pph* 1448A. Furthermore, we identified putative PhoP and PhoQ orthologs, that might be involved in regulation of lipid A modifications. We generated knockouts of all these orthologous genes in *Pph* 1448A and analyzed the impact on the lipid A structure using mass spectrometry. These analyses show that the lipid A remodeling functions of PagL, LpxO, and EptA are conserved in *Pph* 1448A. We also demonstrate that deletion of lipid A remodeling genes does not impair LPS formation and *Pph* 1448A growth kinetics in vitro.

## 2. Results

### 2.1. Orthologues of Lipid A-Modifying Genes Can Be Found in Pseudomonas spp.

**Figure 1:**
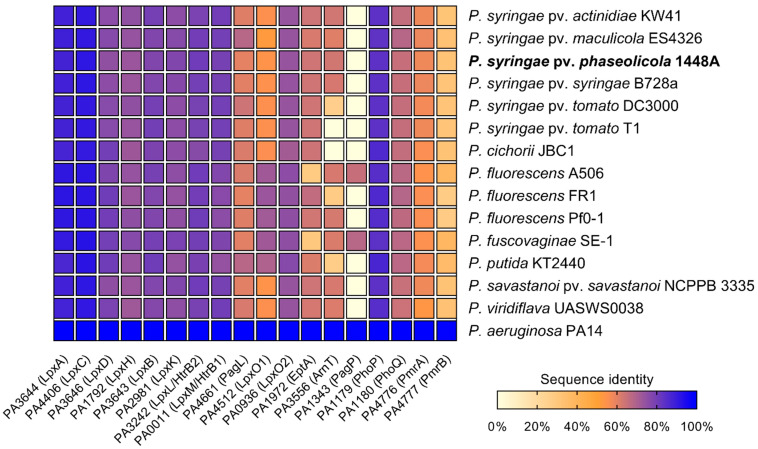
Comparison of sequence identities of lipid A biosynthesis and lipid A-modifying proteins as well as proteins involved in the regulation of lipid A modifications in different *Pseudomonas* species. *P. aeruginosa* PAO1 protein sequences were used as a reference, e-value cutoff = 1 × 10^−4^. Results of the BLASTP analysis are provided in Table S1.

Lipid A biosynthesis as well as lipid A-modifying enzymes are well described in *P. aeruginosa* [11]. Since structural features and dynamic adaption of the lipid A moiety play a crucial role in the host colonization process of *P. aeruginosa*, we were interested in whether orthologous genes are present in different plant-associated *Pseudomonas* spp., which fulfill a similar role. Therefore, predicted proteomes of different publicly available *Pseudomonas* genomes were screened for lipid A biosynthesis and lipid A modification-related protein sequences by BLASTP analysis using the respective protein sequence from *P. aeruginosa* PAO1 as a reference (Figure 1).

In all analyzed *Pseudomonas* spp., proteins involved in lipid A biosynthesis (LpxA, LpxC, LpxD, LpxH, LpxB, LpxK, LpxL, and LpxM) were identified with sequence identities of at least 72% compared to *P. aeruginosa* PAO1, suggesting that the basic lipid A synthesis is conserved in *Pseudomonas*. In contrast, the proteins involved in lipid A modification processes (PagL, LpxO1, LpxO2, EptA, ArnT, PagP) differ considerably in their sequence identity from their orthologs in *P. aeruginosa* in all *Pseudomonas* spp. examined. Whereas putative orthologs of PagL were identified with a protein sequence identity ranging from 59% in *P. fluorescens* FR1 and *Pph* 1448A up to 67% in *P. syringae* pv. *maculicola* ES4326, PagP-mediated palmitoylation seems to be an uncommon feature in plant-associated *Pseudomonas* spp. Of all *Pseudomonas* strains investigated, only *P. fluorescens* A506 and *P. fuscovaginae* seem to have putative orthologs of PagP with sequence identities of 65% and 67%, respectively. Interestingly, all *Pseudomonas* strains analyzed share high sequence identity with LpxO2 from *P. aeruginosa* PAO1, whereas only *P. fluorescens*, *P. fuscovaginae*, and *P. putida* have a second homolog, closely related to LpxO1. A closer look at both identified LpxO proteins in these three strains reveals two different predicted proteins, suggesting that those strains express two proteins for lipid A hydroxylation.

Phosphoethanolamine transferase-like sequences were identified in all *Pseudomonas* spp. except *P*. *fluorescens* A506 and *P. fuscovaginae*, in which the analysis only resulted in hits of approximately 29% sequence identity. This may suggest that neither *P*. *fluorescens* A506 nor P. *fuscovaginae* modify their lipid A with *P*-EtN. Indeed, varying *P*-EtN content has been described in lipid A preparations of different *P*. *fluorescens* strains with some strains lacking almost any *P*-EtN in the lipid A portion [47]. For ArnT, analysis of predicted proteomes of *P. syringae* pv. *tomato* DC3000, *P*. *fluorescens* FR1, and *P*. *putida* K2440 yielded hits with approximately 26% sequence identity, whereas the analysis of other *Pseudomonas* proteomes resulted in hits with identities of 60% or higher. No hit was obtained for an ArnT homolog in *P*. *syringae* pv. *tomato* T1 and *P. cichorii* JBC1.

Furthermore, predicted proteomes of different *Pseudomonas* spp. were searched for the presence of the regulatory TCS PhoP/PhoQ and PmrA/PmrB. PhoP/PhoQ appeared to be conserved among *Pseudomonas* spp., since a sequence identity of at least 83% for PhoP and 64% for PhoQ was determined across all analyzed strains. Analysis of PmrA and PmrB protein sequences yielded often ambiguous and inconclusive hits with low sequence identities. Thus, we could not identify candidates for PmrA and PmrB in the strains studied.

### 2.2. Mass Spectrometric Analysis of Pph 1448A Lipid A Reveals the Activity of PagL, EptA, and LpxO

**Figure 2:**
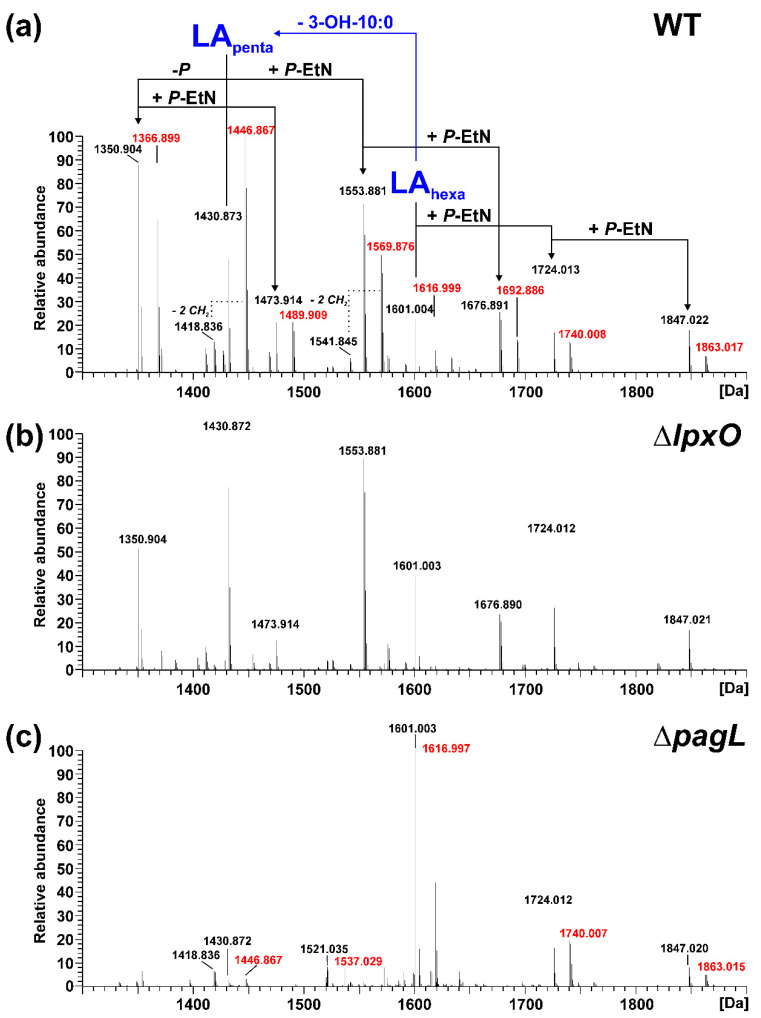
Mass spectrometric analysis of lipid A generated from LPS of the aqueous phase of the phenol-water extract of *Pph* 1448A WT, Δ*lpxO*, and Δ*pagL*. Charge-deconvoluted spectra of the MS analyses performed in negative ion mode are depicted (depicted section: 1300–1900 Da); calculated monoisotopic masses for observed lipid A species are summarized in Table 1; molecular species observed for each strain are listed in Table 2. (**a**) Molecular species distribution in the lipid A preparation of *Pph* 1448A WT, which comprises both penta-(LA_penta_) and hexa-acylated (LA_hexa_) lipid A species, with the penta-acyl species containing one less 3-OH-C10:0 fatty acid. For all major lipid A species, a second species with a mass difference of Δm = 15.995 Da, pointing to the addition of one hydroxyl group, is present. Such peaks are labelled in red. (**b**) These lipid A species are absent from the mass spectrum of the lipid A preparation of *Pph* 1448A Δ*lpxO*. (**c**) The mass spectrum of Δ*pagL* lipid A contains mainly hexa-acylated lipid A species.

**Figure 3:**
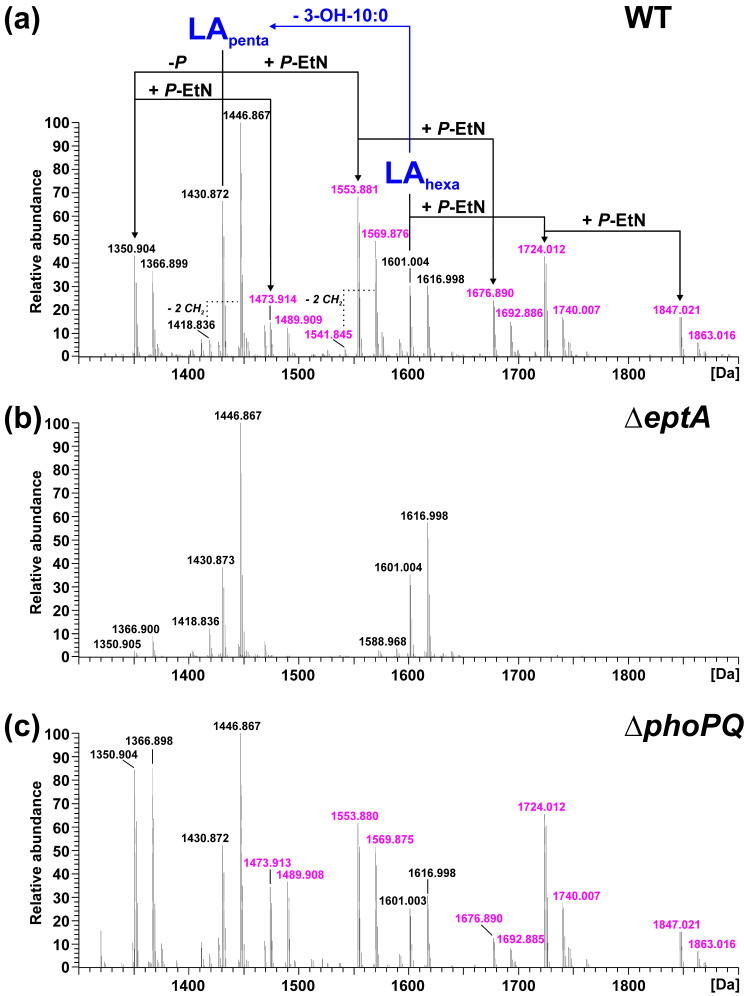
Mass spectrometric analysis of lipid A preparations generated from LPS of the phenolic supernatant of the phenol-water extract of *Pph* 1448A WT, Δ*eptA*, and Δ*phoPQ*. Charge-deconvoluted spectra of MS analyses performed in negative ion mode are depicted (depicted section: 1300–1900 Da); calculated monoisotopic masses for observed lipid A species are summarized in Table 1; molecular species observed for each strain are listed in Table 2. (**a**) Molecular species distribution in the lipid A preparation of *Pph* 1448A WT, which comprises both penta-(LA_penta_) and hexa-acylated (LA_hexa_) lipid A species, with penta-acyl species containing one less 3-OH-C10:0 fatty acid. All lipid A species containing at least one *P*-EtN modification (Δm = 123.009 Da per *P*-EtN) are labelled in pink. (**b**) These *P*-EtN modified lipid A species are absent from the mass spectrum of the lipid A preparation of *Pph* 1448A Δ*eptA*. (**c**) The mass spectrum of Δ*phoPQ* lipid A is comparable to the spectrum of the WT.

We chose *Pph* 1448A, a well characterized and economically relevant plant pathogen, as a model organism for our study of structural modifications of lipid A. To experimentally demonstrate the function of the identified gene loci, we generated isogenic knockout strains of *pagL* (PSPPH_1001), *lpxO* (PSPPH_1567), and *eptA* (PSPPH_1546) in *Pph* 1448A and analyzed their lipid A by MS^1^ and MS^2^ experiments. In general, the observed lipid A pattern showed a high similarity between the aqueous phase and phenolic supernatant LPS preparations of a respective strain. For *Pph* 1448A WT, the mass spectrum of lipid A released from LPS of the aqueous phase is depicted in Figure 2a and the spectrum for lipid A generated from LPS of the phenolic supernatant is shown in Figure 3a.

**Figure 4:**
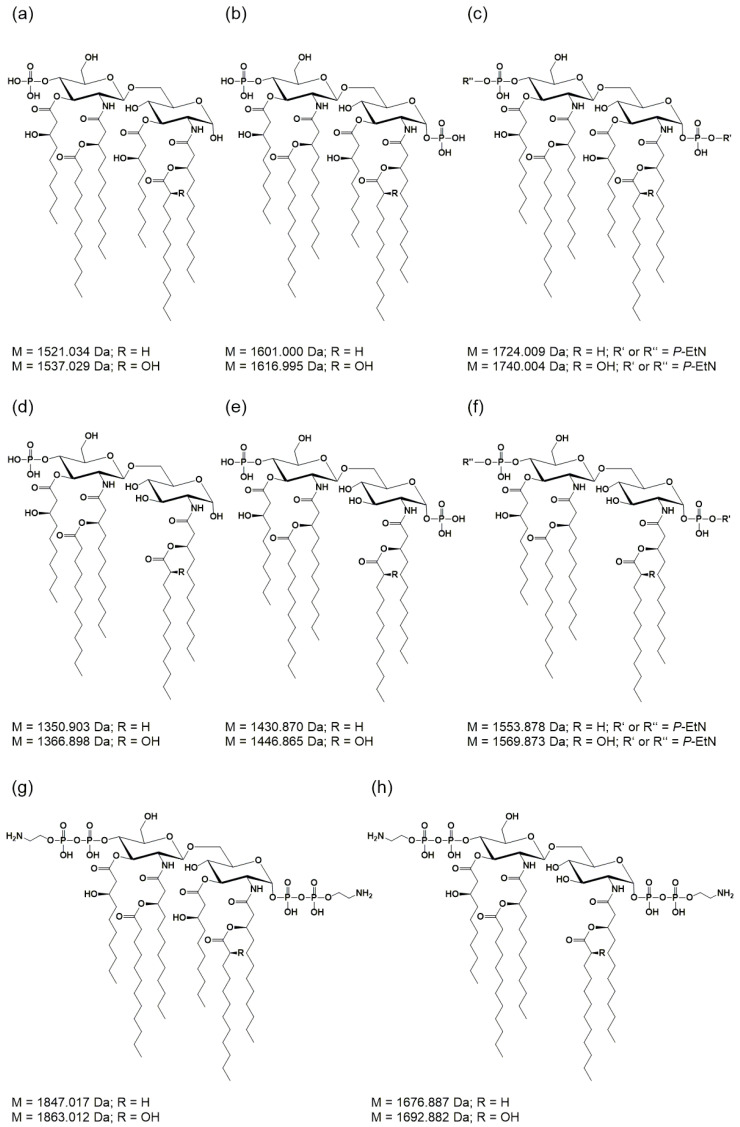
Molecular structures of lipid A species in *Pph* 1448A as determined in this study. (**a**–**c**,**g**) Hexa-acylated lipid A species carrying one or two *P*-EtN substituents or lacking a phosphate. (**d**–**f**,**h**) Penta-acylated lipid A carrying one or two *P*-EtN substituents or lacking a phosphate. M = monoisotopic mass values.

For the lipid A of isogenic mutants, the spectrum comprising the higher number of lipid A species was selected in each case and depicted in comparison to the lipid A of the *Pph* 1448A WT strain from the respective phase. Thus, MS spectra of the lipid A preparations from LPS of the aqueous phase are shown for the Δ*lpxO* (Figure 2b) and the Δ*pagL* (Figure 2c) strain. Mass spectra of lipid A preparations from LPS of the phenolic supernatant are shown for the Δ*eptA* (Figure 3b) and the Δ*phoPQ* (Figure 3c) strain. The different lipid A species identified by MS^1^ analysis of lipid A isolated from *Pph* 1448A WT (Figure 2a and Figure 3a) are summarized in Table 1 and Table 2. They include penta- and hexa-acylated lipid A species present as mono- and di-phosphorylated variants, as well as lipid A species with one or two *P*-EtN modifications. The chemical structures of most of the observed lipid A species are summarized in Figure 4.

**Figure 5:**
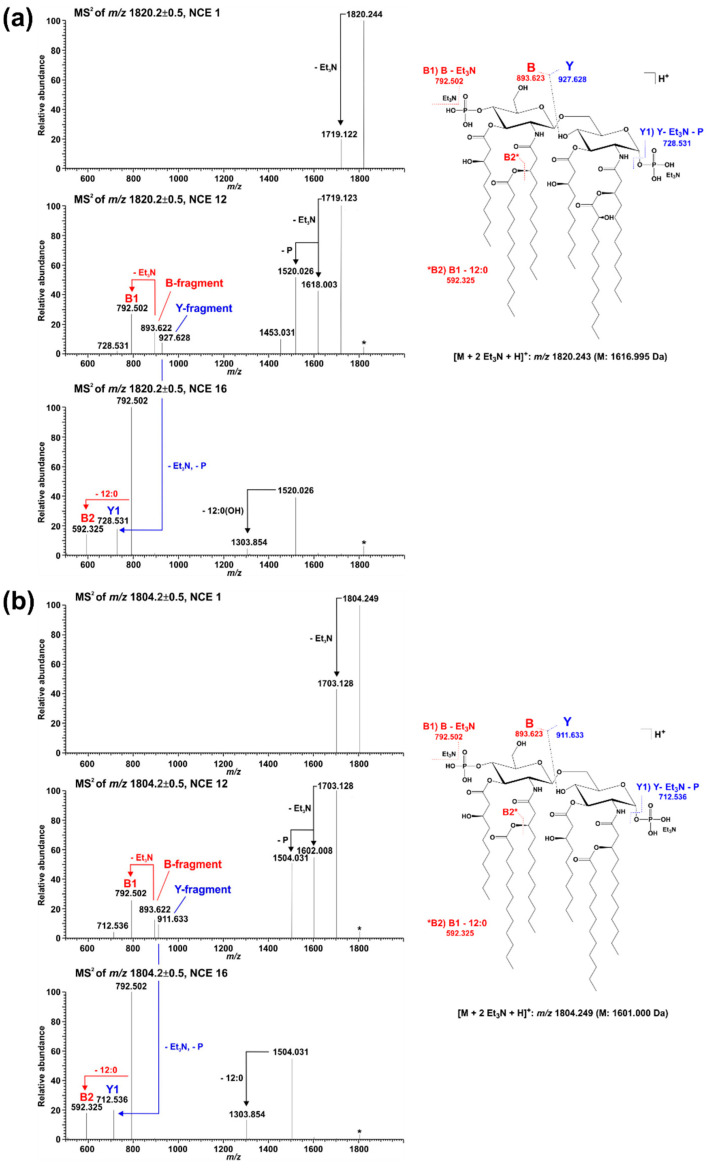
Comparative MS^2^ analysis of the two major di-phosphorylated hexa-acyl lipid A species with a monoisotopic neutral mass of (**a**) 1616.995 Da and (**b**) 1601.000 Da, respectively. For fragmentation analysis, spectra were recorded in a positive ion mode at different NCEs (here depicted in each panel NCE 1 (top), 12 (middle), and 16 (bottom)). Under the selected ionization condition, intensive Et_3_N adduct ions are formed and the respective di-Et_3_N adducts (*m*/*z* 1820.244 in (**a**), *m*/*z* 1804.249 in (**b**)) were selected for MS^2^ experiments. Applying increased NCE leads to the formation of the [M+H]^+^ ion as well as an abundant B-fragment ion (with and without one Et_3_N). At an NCE of 12, the corresponding Y-fragment ion can be detected as well. The presence of the same B-fragment in spectra of both molecules (*m*/*z* 893.622 (with Et_3_N) and *m*/*z* 792.502 (without Et_3_N)) but different Y-fragments (*m*/*z* 927.628 in (**a**), *m*/*z* 911.633 in (**b**)) assigns the hydroxylation event to the secondary C12:0 fatty acid at the amide-bound 3-OH-C12:0 fatty acid of GlcN I.

**Figure 6:**
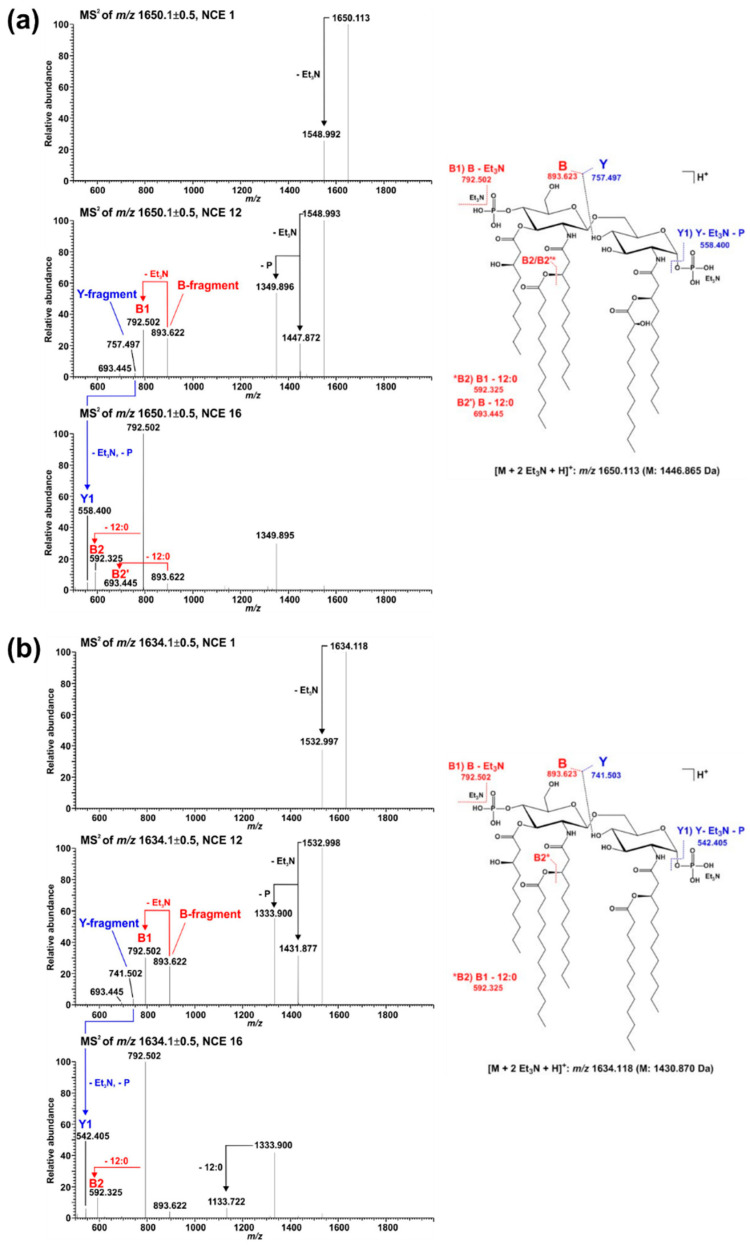
Comparative MS^2^ analysis of the two major di-phosphorylated penta-acyl lipid A species with a monoisotopic neutral mass of (**a**) 1446.865 Da and (**b**) 1430.870 Da, respectively. For fragmentation analysis, spectra were recorded in positive ion mode at different NCEs (here depicted in each panel NCE 1 (top), 12 (middle), and 16 (bottom)). Under the selected ionization condition, intensive Et_3_N adduct ions are formed and the respective di-Et_3_N adducts (*m*/*z* 1650.133 in (**a**), *m*/*z* 1634.118 in (**b**)) were selected for MS^2^ experiments. Applying increased NCE leads to the formation of the [M+H]^+^ ion as well as an abundant B-fragment ion (with and without one Et_3_N). At an NCE of 12, the corresponding Y-fragment ion can be detected as well. The presence of the same B-fragment in spectra of both molecules (*m*/*z* 893.622 (with Et_3_N) and *m*/*z* 792.502 (without Et_3_N)) but different Y-fragments (*m*/*z* 757.497 in (**a**), *m*/*z* 741.502 in (**b**)) assigns the hydroxylation event to the secondary C12:0 fatty acid at the amide-bound 3-OH-C12:0 fatty acid of GlcN I, as shown in Figure 5 for hexa-acylated lipid A as well. Moreover, the Y-fragment ions observed here clearly assign the removal of one 3-OH-C10:0 fatty acid to the *O*-3 position of GlcN I.

Lipid A from *Pph* 1448A WT comprises as main species penta- and hexa-acylated, di-phosphorylated lipid A species at 1430.873 Da and 1601.004 Da, respectively, in line with the typical fatty acid composition of *Pseudomonas* spp. as mentioned in the introduction. In addition, a prominent portion of the penta-acyl lipid A is only mono-phosphorylated (1350.904 Da). For both penta-acyl species, the mono-*P*-EtN substituted (1473.914 Da/1553.881 Da) versions are present; for the di-phosphorylated penta-species the di-*P*-EtN substituted lipid A can be observed as well (1676.891 Da). The same can be seen for the di-phosphorylated hexa-acyl lipid A that can have one (1724.013 Da) or two (1847.022 Da) *P*-EtN moieties in addition. All these eight lipid A species are accompanied by a second species with a mass difference of Δm = 15.995 Da, pointing to a single addition of a hydroxyl group (peaks labelled in red in Figure 2). This hydroxylation is LpxO-dependent (Figure 2b), in line with the proposed function of LpxO as a dioxygenase that hydroxylates secondary fatty acids. These data were further corroborated by GC/MS analysis of the hydroxy fatty acids present in the respective lipid A preparations. Whereas in *Pph* 1448A WT lipid A 3-OH-C10:0, 3-OH-C12:0, and 2-OH-C12:0 fatty acids were detected, the latter was absent from lipid A preparation of the Δ*lpxO* strain (data not shown). Comparative MS^2^ analyses of the di-phosphorylated hexa-acyl (1601.004 Da and 1616.999 Da; Figure 5) and penta-acyl (1430.873 Da and 1446.867 Da; Figure 6) lipid A species proved the sole presence of the secondary 2-OH-C12:0 at the distal glucosamine. Notably, the minor lipid A species with calculated monoisotopic masses of 1418.833 Da and 1541.842 Da (*P*-EtN-modified species of the aforementioned molecule), respectively, can potentially originate from two isomeric molecules that are the result of two different pathways (Table 1), e.g., penta-acyl lipid A with a mass of 1418.833 Da can be synthesized by PagL-mediated removal of a 3-OH-C10:0 fatty acid from the hexa-acylated lipid A with a mass of 1588.964 Da. Alternatively, this penta-acyl lipid A is a likely intermediate of the lipid A biosynthesis carrying only one secondary fatty acid [48].

The presence of penta-acylated lipid A species can be attributed almost completely to the action of PagL. In the mass spectrum of the lipid A preparation from the Δ*pagL* strain (Figure 2c) only a small basal level of such species is observable. The comparative MS^2^ analyses mentioned above enabled us to assign the position of PagL-mediated removal of a 3-OH-C10:0 fatty acid to the *O*-3 position of the distal glucosamine. 

While Figure 2 shows the effects of knocking out genes encoding fatty acid chain-modifying enzymes, Figure 3 focuses on the modification of phosphate residues with *P*-EtN. The *P*-EtN-modified lipid A species observed in *Pph* 1448A WT (Figure 3a) are absent in the Δ*eptA* strain (Figure 3b). Notably, the major mono-phosphorylated penta-acyl lipid A species (1350.904 Da, 1366.899 Da) found in the *Pph* 1448A WT and the other mutant strains are only present in low abundance in this preparation. Interestingly, the mass spectrum obtained for lipid A of the Δ*phoPQ* strain (Figure 3c) is very similar to the *Pph* 1448A WT spectrum (Figure 3a). The fragmentation of *P*-EtN-modified lipid A in MS^2^ experiments is not as indicative as for the di-phosphorylated species. Nonetheless, the mono-*P*-EtN modification seems to be possible on both the 1-*P* or the 4′-*P*, but with a preference for 1-*P* as exemplarily shown for the fragmentation of mono-*P*-EtN substituted, di-phosphorylated penta-acyl lipid A (1553.881 Da; Figure S2). MS^2^ experiments on mono-phosphorylated penta-acyl lipid A (1350.904 Da; Figure S3) revealed that this species lacks the 1-*P*.

For independent verification of the mass spectrometric data and structural alterations, crude lipid A samples generated by a small-scale preparation [49] were analysed by MALDI-TOF (Figure S4).

### 2.3. Lack of Lipid A-Modifying Enzymes Does Not Affect Growth and LPS Formation in Pph 1448A

**Figure 7:**
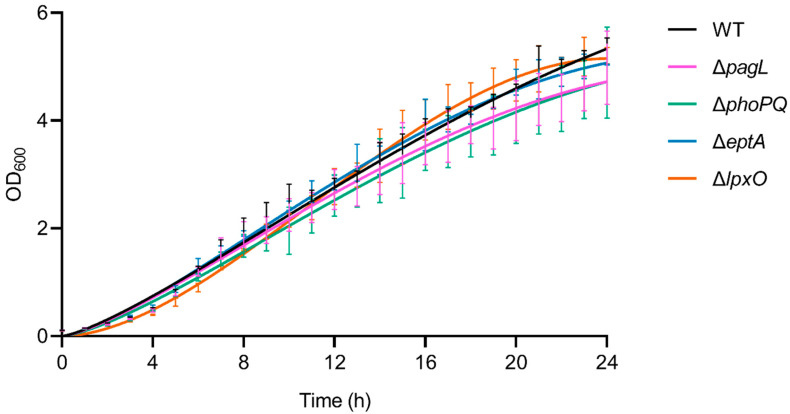
Bacterial growth curves of *Pph* 1448A WT and different mutant strains. Starting with an OD_600_ of 0.1, bacterial growth was monitored hourly over 24 h using a photometer at 600 nm wavelength. No differences in growth kinetics of the mutant strains Δ*pagL*, Δ*phoPQ*, Δ*eptA*, and Δ*lpxO* compared to the WT strain were observed. Each growth curve depicts pooled data from three independent biological replicates. Statistical analysis using multiple *t*-tests comparing the growth of mutant strains hourly to the WT *Pph* 1448A did not show significant differences (*p* > 0.05).

To test whether alterations of the lipid A structure affect growth behavior, we cultivated isogenic derivatives of *Pph* 1448A in liquid medium. All mutant strains showed WT-like growth kinetics (Figure 7). Alterations of the lipid A structure might influence the overall LPS structure. Analysis of *Pph* 1448A LPS via SDS-PAGE showed a similar ladder-like pattern in the mutant strains and the WT (Figure S5). Thus, knockout of lipid A-modifying genes seems not to have major effects on OPS synthesis, LPS size distribution or LPS levels.

## 3. Discussion

This study demonstrates the ability of *P*. *syringae* pv. *phaseolicola* 1448A to remodel its lipid A and identifies the corresponding gene loci of PagL, LpxO, and EptA. We performed mass spectrometric analyses to pinpoint the structural modification for each studied gene. In contrast to the predicted sequences of proteins involved in lipid A biosynthesis, sequences of lipid A-modifying proteins vary to a higher degree in all the strains examined. While LpxO2- and PagL-like protein sequences were found in all strains, the occurrence of LpxO1, EptA, ArnT, and PagP differs among *Pseudomonas* spp.

Palmitoylation of lipid A species alters host innate immune responses, increases resistance to some antimicrobial peptides and facilitates immune evasion of *P. aeruginosa* during colonization of its human host [19]. Interestingly, homologs of *P. aeruginosa* PagP have only been found in *P*. *fluorescens* A506 and *P*. *fuscovaginae* SE-1, yet the role of PagP in these plant-associated bacteria remains unknown. The absence of lipid A palmitoylation in most plant-associated *Pseudomonas* spp. may suggest that this trait is not beneficial for plant colonization or that the energetic costs outweigh the benefits.

We could not identify L-Ara4N additions to the lipid A of *Pph* 1448A by mass spectrometry, although *Pph* 1448A seems to have a homolog of ArnT with 63% sequence identity to *P. aeruginosa* ArnT. Possibly, it may not be expressed under the cultivation conditions used, since ArnT is regulated by PmrA/PmrB in *P. aeruginosa* and induced upon sensing of low pH, high Mg^2+^, Fe^3+^ or Al^3+^ [17,36,50]. Alternatively, the C-terminal truncation of 16 amino acids as compared to *P. aeruginosa* ArnT may render it inactive (Figure S6).

Lipid A analysis of the Δ*lpxO* strain revealed the presence of a lipid A species that lack hydroxylation of secondary fatty acids (Figure 2). Our mass spectrometric data displayed a similar structural phenotype as described for *P. aeruginosa* PAO1 *lpxO*-mutants by Lo Sciuto et al. [14]. In contrast to *P. aeruginosa* PAO1 with two LpxO enzymes, we identified an LpxO2 ortholog in *Pph* 1448A as the only lipid A-hydroxylating enzyme, confirming the results of the predicted proteome analysis. GC/MS-based analysis of hydroxy fatty acids released from the lipid A confirmed the absence of 2-OH-C12:0 fatty acids in the Δ*lpxO* strain, thus further corroborating the role of LpxO in *Pph* 1448A. MS^2^ experiments on lipid A molecules 1616.995 Da and 1446.873 Da, respectively, in comparison with the respective non-hydroxylated species (1601.000 Da/1430.867 Da) show that this LpxO-mediated hydroxylation only takes place on the secondary C12:0 fatty acid at the amide-bound 3-OH-C12:0 fatty acid of GlcN I (distal GlcN; Figure 5 and Figure 6). Thus, PSPPH_1567 encodes the lipid A hydroxylase LpxO in *Pph* 1448A. Like PagL activity, LpxO activity is important for pathogenicity and confers resistance to CAMPs in *P. aeruginosa* [14]. Additionally, *lpxO* mutants in *Acinetobacter baumanii* are less virulent compared to strains expressing a fully hydroxylated lipid A moiety [39]. In *Pph* 1448A, the hydroxylation of lipid A could possibly also support the colonization of its plant host *Phaseolus vulgaris*.

In *P. aeruginosa*, the outer membrane lipase PagL hydrolyses the ester-linked acyl chain at position *O*-3 of lipid A, which results in the release of 3-OH-C10:0 [18]. While *Pph* 1448A synthesizes hexa- and penta-acylated lipid A (Figure 2a and Figure 3a), *Pph* 1448A Δ*pagL* predominantly expresses hexa-acylated lipid A species (Figure 2c). Our results show that the function of PagL (PSPPH_1001) is conserved in *Pph* 1448A. Notably, in order to generate a full length deletion of *pagL*, the overlapping neighboring hypothetical open reading frame of unknown function (PSPPH_1002) was C-terminally truncated (Figure S7). In *Salmonella*, removal of the position *O*-3 acyl chain confers resistance to CAMPs and lowers the affinity for the TLR4/MD2 receptor and enables *Salmonella* to evade immune responses in mammalian hosts [37,51,52]. It is unknown whether *P. vulgaris* recognizes the lipid A moiety of *Pph* 1448A as a microbe-associated molecular pattern (MAMP), which triggers immune responses, and if removal of an acyl chain leads to evasion of plant immunity. Medium-chain 3-hydroxy fatty acids, ranging from C8:0 to C12:0, from *Pseudomonas* trigger immune responses through the cell surface-localized immune receptor LORE in *Arabidopsis thaliana* and other crucifers [15,53]. Interestingly, many plant-associated *Pseudomonas* spp. have orthologs of PagL and presumably release 3-OH-C10:0 fatty acids. However, apparently only *Brassicaceae* evolved a defense mechanism to recognize free medium-chain 3-hydroxy fatty acids [53].

Mass spectra from lipid A of *Pph* 1448A revealed single and double modified phosphate groups carrying *P*-EtN modifications. *P*-EtN modifications were not detected in the respective Δ*eptA* strain (Figure 3b), while all other expected non-hydroxylated and hydroxylated lipid A species were present. Hence, our results show that PSPPH_1546 encodes the phosphoethanolamine transferase EptA in *Pph* 1448A. Orthologous genes of *eptA* can be found in a wide variety of plant-associated Gram-negative bacteria (Figure 1). *P*-EtN additions to the lipid A are mainly used to decrease the overall net negative charge of the molecule [54]. Subsequently, CAMPs are less attracted to the lipid A moiety with a less negative net charge. The overall critical threshold of CAMPs on the bacterial membrane surface is lower and the membrane does not collapse, resulting in the survival of bacteria [55]. EptA-mediated *P*-EtN additions in *Pph* 1448A could help the pathogen to colonize bean plants. Expression of defensin-like antimicrobial peptides against different bacteria has been shown for *Vigna sesquipedalis* [56], for example, and could explain the general importance for *P*-EtN additions to the lipid A in plant-associated *Pseudomonas* spp.

Release of OMVs can be linked to *P*-EtN additions in *C. rodentium* and PagL activity in *Salmonella*. In *C. rodentium*, the release of OMVs is negatively affected by the activity of the two *P*-EtN transferases EptA and CptA, whereas deacylation of lipid A leads to a decrease in the hydrophobic cross-section area of lipid A and promotes OMV release in *Salmonella* [33,38]. Pathogenic Gram-negative bacteria export virulence factors in OMVs to aid the colonization process of the host. However, OMVs also contain MAMPs, which trigger plant immunity and prepare the plant to an upcoming pathogen attack [57,58]. Taken together, *Pph* 1448A may regulate the release of OMV during host colonisation through the addition of *P*-EtN, and PagL might play a role in evading the immune system in plants but may also allow recognition of the pathogen by cruciferous hosts.

Mass spectrometric analysis of lipid A obtained from the WT and from its isogenic mutant strains Δ*lpxO* and Δ*phoPQ* showed significant amounts of dephosphorylated lipid A species (Figure 2 and Figure 3). Gram-negative bacteria are known to dephosphorylate their lipid A via phosphatases. For example, *Helicobacter pylori* utilizes the lipid A 1-phosphatase LpxE and lipid A 4′-phosphatase LpxF to promote its resistance to antimicrobial peptides and secure its survival during host colonization [59,60]. A similar lipid A-modifying process might be utilized by *Pph* 1448A. However, dephosphorylated lipid A species were found when *Pph* 1448A was cultivated in a full-strength medium. Identification of dephosphorylation events during bean plant colonisation could shed light on the relevance of lipid A phosphatases for plant-associated bacteria. Unlike the lipid A of the *Pph* 1448A WT, we identified little to no dephosphorylated lipid A species in the Δ*eptA* mutant. In the absence of *P*-EtN in the outer membrane, the overall net charge was presumably balanced in this way and phosphatase activity was not necessary to rebalance *P*-EtN additions under the growth conditions used. Interplay between lipid A phosphatase activity and *P*-EtN additions may provide pathogens with a strategy to establish itself in an unfavourable environment.

**Figure 8:**
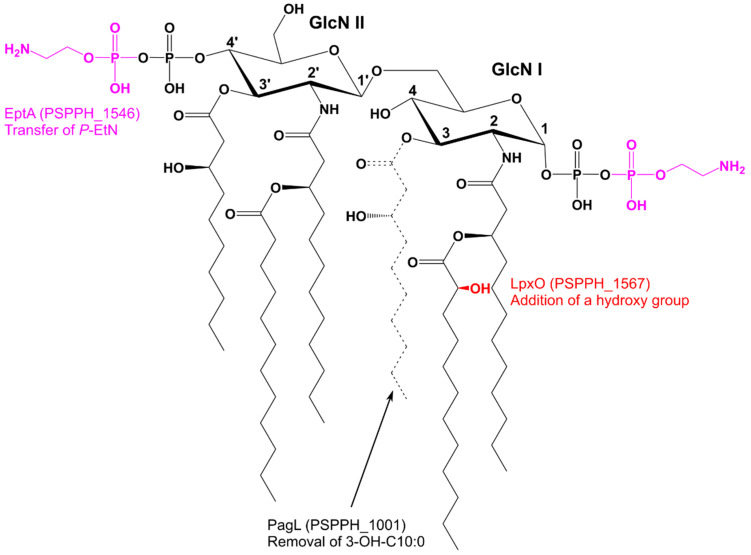
Lipid A modifications identified in *Pph* 1448A. Both of the phosphate groups can be non-stoichiometrically modified by EptA through adding a *P*-EtN. LpxO activity results in the non-stoichiometrical hydroxylation of the secondary acyl chain of the distal glucosamine (GlcN I). PagL removes the 3-OH-C10:0 of the distal glucosamine resulting in penta-acylated lipid A species.

In this study, the detailed characterization of different lipid A modifications (Figure 8) using mass spectrometric analysis was realized, which aids in understanding lipid A remodeling processes in plant-associated *Pseudomonas* spp. Precise annotation of functional group positions on the lipid A moiety enables follow-up studies to examine regulatory processes and modification during host infection. Taken together, our detailed genetic and structural analyses demonstrate the potential of *Pph* 1448A as a model organism to gain insights into the role of lipid A modifications during pathogen–plant interactions.

## 4. Materials and Methods

### 4.1. Strains and Growth Conditions

Strains were grown under shaking at 28 °C for *P. syringae* pv. *phaseolicola* [61] (gifted by John Mansfield, Imperial College London, UK) or 37 °C for *E. coli* strains. Strains used in this study are listed in Table S2. Bacteria were grown in King’s B medium (KB) [62], or Lysogeny Broth [63]. Antibiotics were used at a final concentration of gentamicin at 5 µg mL^−1^, rifampicin at 50 µg mL^−1^, kanamycin at 25 µg mL^−1^. To identify positive *E. coli* clones X-gal and IPTG were added to growth media at 30 µg mL^−1^ and 0.05 mM, respectively.

### 4.2. Analysis of Predicted Proteomes of Pseudomonas spp.

Predicted proteomes of different *Pseudomonas* spp. were analyzed to determine amino acid sequence identity using the BLASTP function on pseudomonas.com (access date 7 December 2021). Cut-off was set to 1 × 10^-4^, word size 3, filtered and no pairwise output. If multiple hits were obtained in a single predicted proteome, the sequence identity of the best hit according to the expected cut-off value and the bit score were used and depicted in the Table S1 and Figure 1.

### 4.3. Gene Knockout in Pph 1448A

*Pph* 1448A knockout mutants were generated using the pGGKO-blue plasmid as described previously with minor changes [64]. In brief, flanking sequences (450 to 650 bp) of target genes were amplified via PCR from genomic DNA and inserted into pGGKO-blue backbone using Golden-gate cloning. Competent *E. coli* DH5α [65] cells were transformed with this precursor plasmid. Precursor plasmid was isolated and a Gm^R^ resistance cassette was inserted between the flanking sequences. Final plasmids were verified by Sanger sequencing. Mutants of *Pph* 1448A were generated as described with minor changes [66]. Triparental mating conjugation with *E. coli* HB101 [67] as a helper strain was used to transfer the plasmids into *Pph* 1448A. Positive recombinants were counter selected on KB Rif Gent Sucrose and insertion of the Gm^R^ resistance cassette verified by Sanger sequencing of the respective gene loci (Figures S7–S10). Oligonucleotides used in this study are listed in Table S3.

### 4.4. Preparation of Lipopolysaccharide from Pph 1448A Strains

*Pph* 1448A strains were cultivated overnight under shaking and harvested via centrifugation (4 °C, 8000× *g*, 20 min) at an OD_600_ = 1.2 to 1.5. Bacterial pellets were washed two times with pre-cooled ddH_2_O prior to freeze drying. Freeze-dried bacterial pellets were dissolved in 100% EtOH and the suspension was stirred for 2 h at room temperature. The suspension was filtered (Whatman 595 ½) and sequentially washed with acetone (twice) and diethyl ether. Washed pellets were dried and resuspended in water (15 mg mL^−1^). 10% NaN_3_ was added to a final concentration of 0.02% to the dissolved pellets. Dissolved pellets were sequentially treated with DNase/RNase (10 mg mL^−1^ *w*/*v*) and proteinase K (10 mg mL^−1^ *w*/*v*) at room temperature (100 µL enzyme per g dry weight). Digested pellets were dialyzed (14 kDa cut-off) for 2 days in ddH_2_O and freeze dried.

For LPS isolation, the hot phenol-water method was used [68]. In brief, dried dialysates were dissolved in pre-warmed ddH_2_O (68 °C, 100 mL per 10 g dry weight) until dialysates were completely dissolved. 90% aqueous phenol (equal volume as ddH_2_O) was added and stirred for 30 min at 68 °C. Aqueous-phenol suspensions were centrifuged (5600× *g*, 4 °C, 20 min) and the upper aqueous phase was collected. The extraction was repeated with the same amount of ddH_2_O that had been collected. Combined aqueous and phenolic phases were dialyzed against deionized water separately. Phenolic phases were separated via centrifugation (600× *g*, 20 °C, 5 min) into supernatant and phenolic pellet before freeze-drying.

### 4.5. Lipid A Preparation

Lipid A was prepared from LPS of the aqueous phase and the phenolic supernatant as described [53]. Briefly, LPS was dissolved in water (7.5 mg mL^−1^) and a solution of 10% SDS (volume equivalent to 12.5% of the water volume) as well as the same volume of acetate buffer (1 M NaOAc, pH 4.4) were added. The mixture was heated for 3 h at 100 °C under slight stirring and freeze-dried. SDS was removed by four washes with 30 mL 2 M HCl/EtOH (1:99 *v*/*v* (6000× *g* for 20 min at 20 °C)). The dried pellet was resuspended in 4 mL water. Afterwards, 4 mL CHCl_3_ and CH_3_OH (4:1 *v*/*v*) were added and the suspension was mixed vigorously and centrifuged (6000× *g*) for 10 min at 4 °C. The organic phase was collected and the water phase (including the interphase) was extracted again three times with 3 mL CHCl_3_. All organic phases were combined and dried under a stream of nitrogen.

### 4.6. Mass Spectrometric Analysis of Lipid A via ESI-MS

All mass spectrometric analyses of lipid A preparations were performed on a Q Exactive Plus (ThermoFisher Scientific, Bremen, Germany) using a Triversa Nanomate (Advion, Ithaca, NY, USA) as nano-ESI source. Lipid A extracts were initially dissolved in a concentration of 5 µg µL^−1^ in chloroform:methanol:water (60:30:4.5 *v*/*v*/*v*). 5 µL of this solution were mixed with 95 µL of water/propan-2-ol/7 M triethylamine/acetic acid (50:50:0.06:0.02 *v*/*v*/*v*/*v*). Mass spectra were recorded for 0.50 min in the negative mode in an *m*/*z*-range of 400–2500 applying a spray voltage of −1.1 kV. All depicted MS^1^ spectra were charge deconvoluted (Xtract module of Xcalibur 3.1 software (ThermoFisher Scientific, Bremen, Germany)) and all provided values refer to the monoisotopic mass of neutral molecules.

To further investigate the lipid A structure, MS^2^ experiments were performed using 5 µL of the above mentioned lipid A solutions mixed with 10 µL chloroform:methanol:water (60:30:4.5 *v*/*v*/*v*) and 285 μL water/propan-2-ol/30 mM ammonium acetate/acetic acid mixture (15:15:1:0.04 *v*/*v*/*v*/*v*). Shortly before analysis, 0.5 μL triethylamine were added and the mixture was thoroughly mixed. Single charged ions of interest were selected and spectra were recorded in a positive ion mode at normalized collision energies (NCE) of 1, 10, 12, 14, 16, 20, and 30. Under these ionization condition, intensive Et_3_N adduct ions are formed. Usually, the di-Et_3_N adducts were selected for MS^2^ experiments, only for mono-phosphorylated lipid A, the mono-Et_3_N adduct was selected. Applying increased NCE leads to the formation of the [M+H]^+^ ion as well as the abundant B-fragment (according to the nomenclature of [69]). At an NCE of 12 or 14, the corresponding Y-fragment ion can usually be well detected, especially for di-phosphorylated lipid A species. Depending on the individual fragmentation of the investigated lipid A species, MS^2^ spectra of NCE values with highest structural information content were selected for representation. The general distribution of 3-OH-acyl chains in *Pseudomonas* lipid A molecules is assigned according to and in line with earlier published structural analysis [70].

### 4.7. Analysis of Fatty Acids via GC-MS

The nature of hydroxy fatty acids in lipid A preparations of *Pph* 1448A WT and Δ*lpxO* was determined by generating the trimethylsilyl (TMS) derivatives of the respective FAMEs in comparison to authentic standards. The gas–liquid chromatography–mass spectrometry (GLC-MS) analyses were performed on an Agilent Technologies 6890N gas chromatograph coupled to a 5975 inert XL Mass Selective Detector (Agilent Technologies Santa Clara, CA, USA). A 30-m Agilent J&W DB-WAX Ultra Inert column (0.25 mm inner diameter, 0.25 µm film thickness) was used and a temperature gradient starting at 70 °C (kept for 1.5 min), then raised at 60 °C/min to 150 °C, kept there for 5 min, and raised to 220 °C at 1.5 °C/min was applied.

### 4.8. Crude LPS Extraction

LPS was prepared from 2 mL bacterial overnight cultures using the protocol from Hitchcock and Brown [71] with minor changes. Bacterial cells were harvested via centrifugation (2000× *g*, 20 °C, 5 min) and the pellets were resuspended in 1 mL 0.15 M NaCl. The suspension was centrifuged at 10000× *g* for 10 min (20 °C) and the supernatant was discarded. The pellet was resuspended in 1 mL lysing buffer (2% SDS (*v*/*v*), 8% β-mercaptoethanol (*v*/*v*), 10% glycerol (*v*/*v*), 1 M Tris (pH 6.8, *v*/*v*) and 0.02% bromphenol blue (*v*/*v*)) and incubated at 100 °C for 10 min. 200 µL Proteinase K (10 mg mL^−1^) were added and samples were incubated at 60 °C for 1 h.

### 4.9. Mass Spectrometric Analysis of Crude Lipid A Extracts via MALDI-TOF

Freeze-dried bacterial pellets were rehydrated with endotoxin-free water, vortexed, then pelleted. Supernatant was discarded. Lipid A was extracted from cell pellets using an ammonium hydroxide-isobutyric acid-based procedure [49,72]. Briefly, a bacterial cell pellet was resuspended in 400 μL of 70% isobutyric acid (Sigma-Aldrich, I1754-1L) and 1 M ammonium hydroxide (Sigma-Aldrich, St. Louis, MO, USA, 221228-500ML-A) (5:3 *v*/*v*). Samples were incubated for 1 h at 100 °C and centrifuged at 8000× *g* for 5 min. Supernatants were collected, added to endotoxin-free water (1:1 *v*/*v*), snap-frozen on dry ice, and lyophilized overnight. The resultant material was washed twice with 1 mL methanol (Fisher Scientific, Waltham, MA, USA, A456-1), and lipid A was extracted using 80 μL of a mixture of chloroform (Fisher Scientific, C606SK-4), methanol, and water (3:1:0.25 *v*/*v*/*v*). Once extracted, 1 μL of the concentrate was spotted on a steel re-usable MALDI plate (Hudson Surface Technology, Closter, NJ, USA, PL-PD-000040-P) followed by 1 μL of 10 mg mL^−1^ norharmane matrix (Sigma-Aldrich, NG252-1G) in chloroform-methanol (2:1 *v*/*v*) (Sigma-Aldrich, St. Louis, MO, USA) and was then air dried. All samples were analyzed on a Bruker Microflex mass spectrometer (Bruker Daltonics, Billerica, MA, USA) in the negative-ion mode with reflection mode. An electrospray tuning mix (Agilent Technologies, Foster City, CA, USA, G2421A) was used for mass calibration. Spectral data were analyzed with Bruker Daltonics FlexAnalysis software (v4.30). The resulting spectra were used to estimate the lipid A structures present in each strain based on their predicted structures and molecular weights.

### 4.10. SDS-PAGE and Silver Staining of Bacterial LPS

2.5 µL of crude LPS sample was mixed with 5 µL of NuPAGE™ LDS Sample Buffer (Invitrogen, Carlsbad, CA, USA, NP0007), NuPAGE™ Sample Reducing Agent (Invitrogen, NP0004) and 15.5 µL ddH_2_O. Samples were incubated at 70 °C for 10 min prior to loading on the gel. LPS samples were separated using NuPAGE™ 4 to 12%, Bis-Tris, 1.0 mm, Mini Protein Gel (Invitrogen, NP0321) with MES as running buffer at 200 V constant settings for 40 min. After separation, the gel was kept for 2 h in fixing solution (30% *v*/*v*, EtOH, 10% *v*/*v* acetic acid), transferred into oxidizing solution (7% *v*/*v* periodic acid, 30% *v*/*v* EtOH, 10% *v*/*v* acetic acid) and washed three times with ddH_2_O. The gel was incubated in dye solution (0.1% *w*/*v* AgNO_3_ in ddH_2_O) in the dark for 30 min before revealing signals on the gel with the revealer solution (3% *w*/*v* Na_2_CO_3_, 0.02% *v*/*v* formaldehyde) for 10 min in the dark. Coloration was stopped with 1% *v*/*v* acetic acid aqueous solution; the gel was washed three times with ddH_2_O for 10 min and photographed. Three independent biological replicates were carried out, showing the same pattern on the gel.

### 4.11. Bacterial Growth Curve

Bacteria were grown overnight in KB supplemented with respective antibiotics. Starting OD_600_ of main cultures was adjusted to 0.1 in KB and the samples incubated at 28 °C with shaking. OD_600_ was monitored every hour over 24 h. Three independent biological replicates were carried out and statistical analysis was assessed using multiple *t*-tests (alpha = 0.05, Holm–Sidak method) comparing the bacterial growth of the mutant strains to the WT every hour.

## Figures and Tables

**Table 1:** Summary of calculated monoisotopic neutral masses of lipid A species identified in *Pph* 1448A including the assignment of specific positions of primary and secondary fatty acids as well as phosphate and phosphoethanolamine modifications. Experimentally determined monoisotopic neutral masses are summarized in Table 2. *P* = monophosphate, *PP*-EtN = diphosphate ethanolamine.

Calculated Mono-Isotopic Mass	Substituting Acyl or Acyloxy-Acyl Chains/Additional Residues on Lipid A Backbone					
[Da]	C-4′	C-3′	C-2′	C-3	C-2	C-1
1322.872	*P*	10:0((*R*)-3-OH)	12:0((*R*)-3-O(12:0)) ^#^	H	10:0((*R*)-3-O(12:0)) ^#^	
1338.867	*P*	10:0((*R*)-3-OH)	12:0((*R*)-3-O(12:0)) ^#^	H	10:0((*R*)-3-O(12:0((*S*)-2-OH))) ^#^	
1350.903	*P*	10:0((*R*)-3-OH)	12:0((*R*)-3-O(12:0))	H	12:0((*R*)-3-O(12:0))	
1366.898	*P*	10:0((*R*)-3-OH)	12:0((*R*)-3-O(12:0))	H	12:0((*R*)-3-O(12:0((*S*)-2-OH)))	
1402.838	*P*	10:0((*R*)-3-OH)	12:0((*R*)-3-O(12:0)) ^#^	H	10:0((*R*)-3-O(12:0)) ^#^	*P*
1418.833 ^§^	*P*	10:0((*R*)-3-OH)	12:0((*R*)-3-O(12:0)) ^#^	H	10:0((*R*)-3-O(12:0((*S*)-2-OH))) ^#^	*P*
1418.833 ^§^	*P*	10:0((*R*)-3-OH)	12:0((*R*)-3-OH) ^##^	10:0((*R*)-3-OH)	12:0((*R*)-3-O(12:0)) ^##^	*P*
1430.870	*P*	10:0((*R*)-3-OH)	12:0((*R*)-3-O(12:0))	H	12:0((*R*)-3-O(12:0))	*P*
1446.865	*P*	10:0((*R*)-3-OH)	12:0((*R*)-3-O(12:0))	H	12:0((*R*)-3-O(12:0((*S*)-2-OH)))	*P*
1473.912	*PP*-EtN	10:0((*R*)-3-OH)	12:0((*R*)-3-O(12:0))	H	12:0((*R*)-3-O(12:0))	
1489.907	*PP*-EtN	10:0((*R*)-3-OH)	12:0((*R*)-3-O(12:0))	H	12:0((*R*)-3-O(12:0((*S*)-2-OH)))	
1521.034	*P*	10:0((*R*)-3-OH)	12:0((*R*)-3-O(12:0))	10:0((*R*)-3-OH)	12:0((*R*)-3-O(12:0))	
1537.029	*P*	10:0((*R*)-3-OH)	12:0((*R*)-3-O(12:0))	10:0((*R*)-3-OH)	12:0((*R*)-3-O(12:0((*S*)-2-OH)))	
1541.842 ^§§^	*P* *	10:0((*R*)-3-OH)	12:0((*R*)-3-O(12:0)) ^#^	H	10:0((*R*)-3-O(12:0((*S*)-2-OH))) ^#^	*PP*-EtN *
1541.842 ^§§^	*P* *	10:0((*R*)-3-OH)	12:0((*R*)-3-OH) ^##^	10:0((*R*)-3-OH)	12:0((*R*)-3-O(12:0)) ^##^	*PP*-EtN *
1553.878	*P* *	10:0((*R*)-3-OH)	12:0((*R*)-3-O(12:0))	H	12:0((*R*)-3-O(12:0))	*PP*-EtN *
1569.873	*P* *	10:0((*R*)-3-OH)	12:0((*R*)-3-O(12:0))	H	12:0((*R*)-3-O(12:0((*S*)-2-OH)))	*PP*-EtN *
1572.969	*P*	10:0((*R*)-3-OH)	12:0((*R*)-3-O(12:0)) ^#^	10:0((*R*)-3-OH)	10:0((*R*)-3-O(12:0)) ^#^	*P*
1588.964	*P*	10:0((*R*)-3-OH)	12:0((*R*)-3-O(12:0)) ^#^	10:0((*R*)-3-OH)	10:0((*R*)-3-O(12:0((*S*)-2-OH))) ^#^	*P*
1601.000	*P*	10:0((*R*)-3-OH)	12:0((*R*)-3-O(12:0))	10:0((*R*)-3-OH)	12:0((*R*)-3-O(12:0))	*P*
1616.995	*P*	10:0((*R*)-3-OH)	12:0((*R*)-3-O(12:0))	10:0((*R*)-3-OH)	12:0((*R*)-3-O(12:0((*S*)-2-OH)))	*P*
1676.887	*PP*-EtN	10:0((*R*)-3-OH)	12:0((*R*)-3-O(12:0))	H	12:0((*R*)-3-O(12:0))	*PP*-EtN
1692.882	*PP*-EtN	10:0((*R*)-3-OH)	12:0((*R*)-3-O(12:0))	H	12:0((*R*)-3-O(12:0((*S*)-2-OH)))	*PP*-EtN
1724.009	*P* *	10:0((*R*)-3-OH)	12:0((*R*)-3-O(12:0))	10:0((*R*)-3-OH)	12:0((*R*)-3-O(12:0))	*PP*-EtN *
1740.004	*P* *	10:0((*R*)-3-OH)	12:0((*R*)-3-O(12:0))	10:0((*R*)-3-OH)	12:0((*R*)-3-O(12:0((*S*)-2-OH)))	*PP*-EtN *
1847.017	*PP*-EtN	10:0((*R*)-3-OH)	12:0((*R*)-3-O(12:0))	10:0((*R*)-3-OH)	12:0((*R*)-3-O(12:0))	*PP*-EtN
1863.012	*PP*-EtN	10:0((*R*)-3-OH)	12:0((*R*)-3-O(12:0))	10:0((*R*)-3-OH)	12:0((*R*)-3-O(12:0((*S*)-2-OH)))	*PP*-EtN

^#^ length of amide-bound 3-OH-fatty acid might be interchanged; ^##^ position of secondary fatty acid might be interchanged; * position of *P* and *PP*-EtN can be interchanged, but this is the predominant species (see Figure S2); ^§,§§^ isomers originating from different pathways.

**Table 2:** Calculated and experimentally determined monoisotopic masses of lipid A species observed in MS^1^ spectra shown in Figure 2 and Figure 3. Annotation accuracy of chemical structures to mass measurements are stated as Δppm. For more detailed structural information see Table 1 and Figure 4.

	Strain (Figure Number of Respective MS^1^ Spectrum)											
	WT (2a)		Δ*lpxO* (2b)		Δ*pagL* (2c)		WT (3a)		Δ*eptA* (3b)		Δ*phoPQ* (3c)	
M_cal_	M_exp_*	Error (Δppm)	M_exp_*	Error (Δppm)	M_exp_*	Error (Δppm)	M_exp_*	Error (Δppm)	M_exp_*	Error (Δppm)	M_exp_*	Error (Δppm)
1322.872	*1322.872*	0.0	*1322.872*	0.0	n.d.	-	*1322.872*	0.0	n.d.	-	*1322.871*	−0.8
1338.867	*1338.867*	0.0	n.d.	-	*1338.866*	−0.7	*1338.868*	0.7	*1338.869*	1.5	*1338.866*	−0.7
1350.903	1350.904	0.7	1350.904	0.7	n.d.	-	1350.904	0.7	*1350.905*	1.5	1350.904	0.7
1366.898	1366.899	0.7	n.d.	-	*1366.898*	0.0	1366.899	0.7	*1366.900*	1.5	1,366.898	0.0
1402.838	n.d. **	-	*1402.841*	2.1	n.d.	-	*1402.842*	2.9	*1402.842*	2.9	n.d.	-
1418.833 ^$^	1418.836	2.1	*1418.837*	2.8	*1418.836*	2.1	*1418.836*	2.1	1,418.836	2.1	*1418.836*	2.1
1430.870	1430.873	2.1	1430.872	1.4	*1430.872*	1.4	1430.872	1.4	1,430.873	2.1	1430.872	1.4
1446.865	1446.867	1.4	n.d.	-	*1446.867*	1.4	1446.867	1.4	1,446.867	1.4	1446.867	1.4
1473.912	1473.914	1.4	1473.914	1.4	n.d.	-	1473.914	1.4	n.d.	-	1473.913	0.7
1489.907	1489.909	1.3	n.d.	-	n.d.	-	1489.909	1.3	n.d.	-	1489.908	0.7
1521.034	*1521.034*	0.0	*1521.035*	0.7	*1521.035*	0.7	*1521.034*	0.0	n.d.	-	*1521.033*	−0.7
1537.029	*1537.029*	0.0	n.d.	-	*1537.029*	0.0	*1537.030*	0.7	*1537.031*	1.3	*1537.029*	0.0
1541.842 ^$^	*1541.845*	2.0	*1541.846*	2.6	*1541.845*	2.0	*1541.845*	2.0	n.d.	-	n.d.	-
1553.878	1553.881	1.9	1553.881	1.9	*1553.882*	2.6	1553.881	1.9	n.d.	-	1553.880	1.3
1569.873	1569.876	1.9	n.d.	-	*1569.877*	2.5	1569.876	1.9	n.d.	-	1569.875	1.3
1572.969	n.d.	-	n.d.	-	*1572.972*	1.9	*1572.973*	2.5	*1572.973*	2.5	n.d.	-
1588.964	n.d.	-	n.d.	-	*1588.967*	1.9	*1588.968*	2.5	*1588.968*	2.5	n.d.	-
1601.000	1601.004	2.5	1601.003	1.9	1601.003	1.9	1601.004	2.5	1601.004	2.5	1601.003	1.9
1616.995	1616.999	2.5	n.d.	-	1616.997	1.2	1616.998	1.9	1616.998	1.9	1616.998	1.9
1676.887	1676.891	2.4	1676.890	1.8	n.d.	-	1676.890	1.8	n.d.	-	1676.890	1.8
1692.882	1692.886	2.4	n.d.	-	n.d.	-	1692.886	2.4	n.d.	-	*1692.885*	1.8
1724.009	1724.013	2.3	1724.012	1.7	1724.012	1.7	1724.012	1.7	n.d.	-	1724.012	1.7
1740.004	1740.008	2.3	n.d.	-	1740.007	1.7	1740.007	1.7	n.d.	-	1740.007	1.7
1847.017	1847.022	2.7	1847.021	2.2	*1847.020*	1.6	1847.021	2.2	n.d.	-	1847.021	2.2
1863.012	1863.017	2.7	n.d.	-	*1863.015*	1.6	*1863.016*	2.1	n.d.	-	*1863.016*	2.1

* minor species (<10% of relative intensity compared to the most intensive lipid A molecule) are stated in italic style; ** n.d. = not detected; ^$^ two different lipid A species with this exact molecular mass can be present (compare Table 1).

## Data Availability

All data supporting the findings of this study are provided in the manuscript and its Supplementary Files. Additional data supporting the findings of this study are available from the corresponding authors upon request.

## Supplementary Materials

The following supporting information can be downloaded at: https://www.mdpi.com/article/10.3390/ijms23041996/s1.
