# Supplementary material for: Remodeling of Lipid A in Pseudomonas syringae pv. phaseolicola In Vitro
Source: Int J Mol Sci. 2022 Feb 11;23(4):1996. doi: 10.3390/ijms23041996 (PMC8876380; doi:10.3390/ijms23041996)
Supplement: Supplementary file 1 [file ijms-23-01996-s001.zip › ijms-1557874-supplementary.pdf]

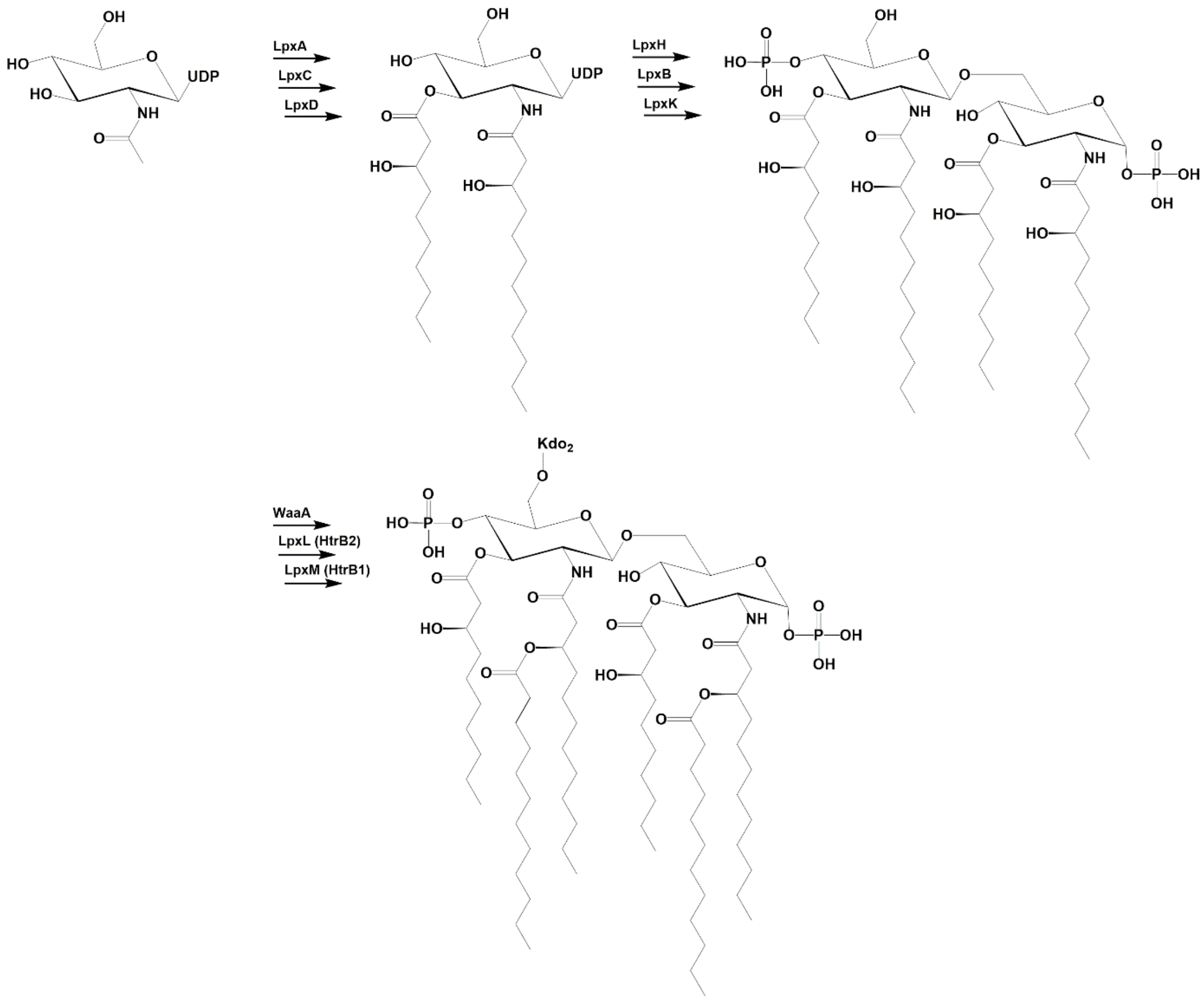

Figure S1. Lipid A biosynthesis in *Pseudomonas aeruginosa*. The UDP-GlcNAc 3-*O*-acyltransferase LpxA catalyzes the addition of a 3-OH-C10:0 to the uridine diphosphate *N*-acetylglucosamine (UDP-D-GlcNAc). Next, LpxC deacetylates the UDP-D-GlcNAc, followed by the addition of a 3-OH-C12:0 catalyzed by the 3-*O*-acyl-GlcN 2-*N*-acyltransferase LpxD. The UDP-diacyl-GlcN pyrophosphatase LpxH forms a mono-phosphorylated GlcN. LpxB, a disaccharide synthase, catalyzes the condensation of the mono-phosphorylated GlcN and a UDP-2,3-diacyl-GlcN. LpxK phosphorylates the 4′-position of the resulting tetraacyl-β-(1→6)-di-GlcN monophosphate. WaaA catalyzes the addition of 3-deoxy-D-*manno*-oct-2-ulosonic acid (Kdo) moieties to the distal GlcN. The secondary lauroyl 2-hydroxylases LpxL (HtrB2) and LpxM (HtrB1) catalyze the addition of C12:0 chains to the amide-linked primary 3-OH-C12:0 acyl chains.

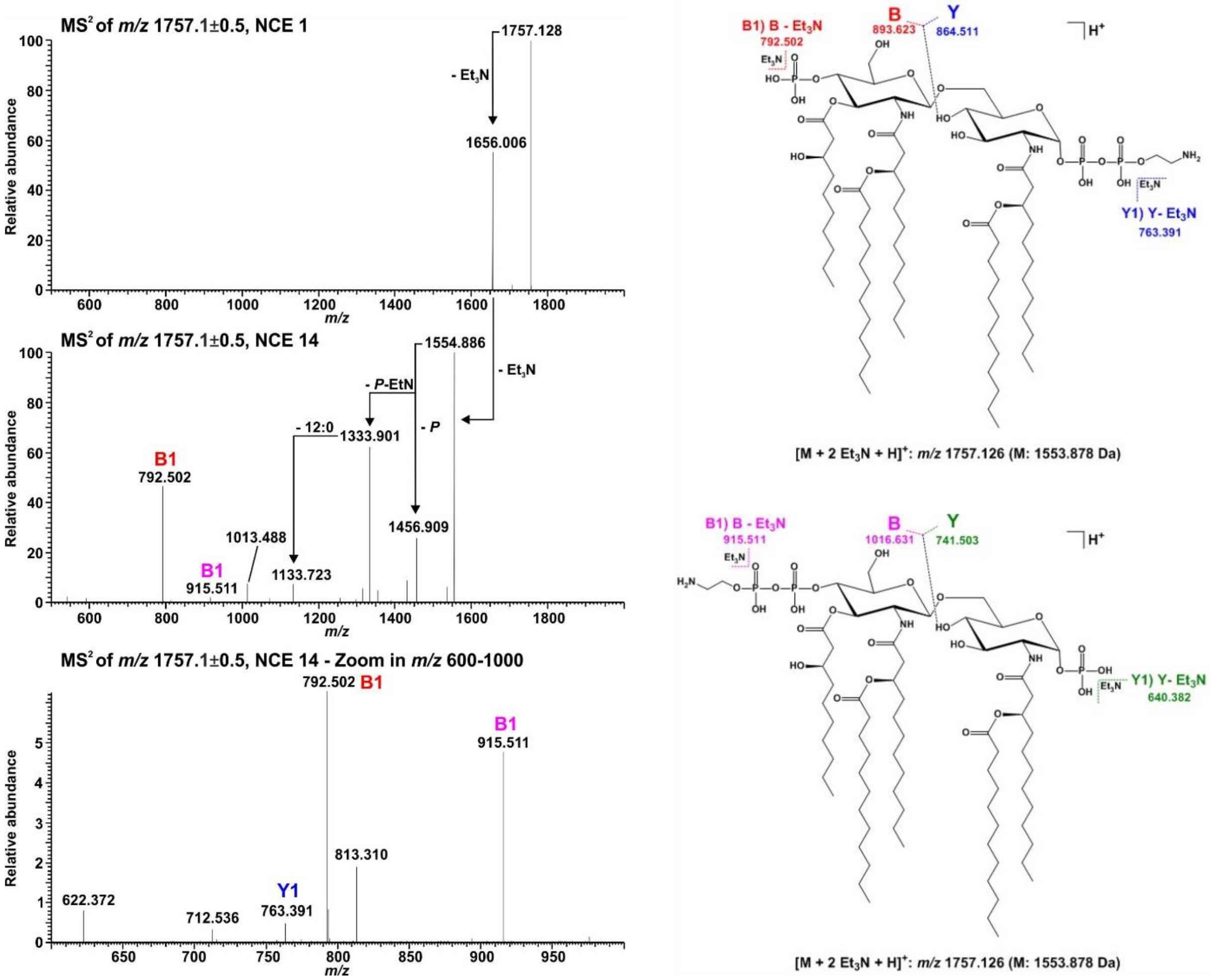

Figure S2. MS^2 analysis of the penta-acylated, di-phosphorylated and mono-*P*-EtN substituted lipid A species with a monoisotopic neutral mass of 1553.878 Da, analogously performed as experiments shown in Figures 5 and 6. The formed di-Et_3N adduct has been selected for the MS^2 experiment (*m/z* 1757.128; top panel). Since both the 1-*P* as well as the 4′-*P* can potentially be *P*-EtN modified both possible structures, including respective putative fragment masses, are depicted on the right side. Notably, the presence of *P*-EtN changes the fragmentation behaviour in such a way that only fragments without Et_3N addition are observable. Both possible B-fragment ions can be found in the spectrum recorded with an NCE of 14, with the one of the lipid A carrying the *P*-EtN on the 1-*P* (*m/z* 792.502; B1) being much more prominent than the one of the lipid A carrying the *P*-EtN on the 4′-*P* (*m/z* 915.511; B1) (middle panel). For the first species, the corresponding Y-fragment without Et_3N addition (Y1; *m/z* 763.391) can be observed in a very tiny proportion when zooming into the spectrum (bottom panel), whereas the Y1 (calc. *m/z* 640.382) is not observable, most likely due to the much lower abundance of this lipid A variant.

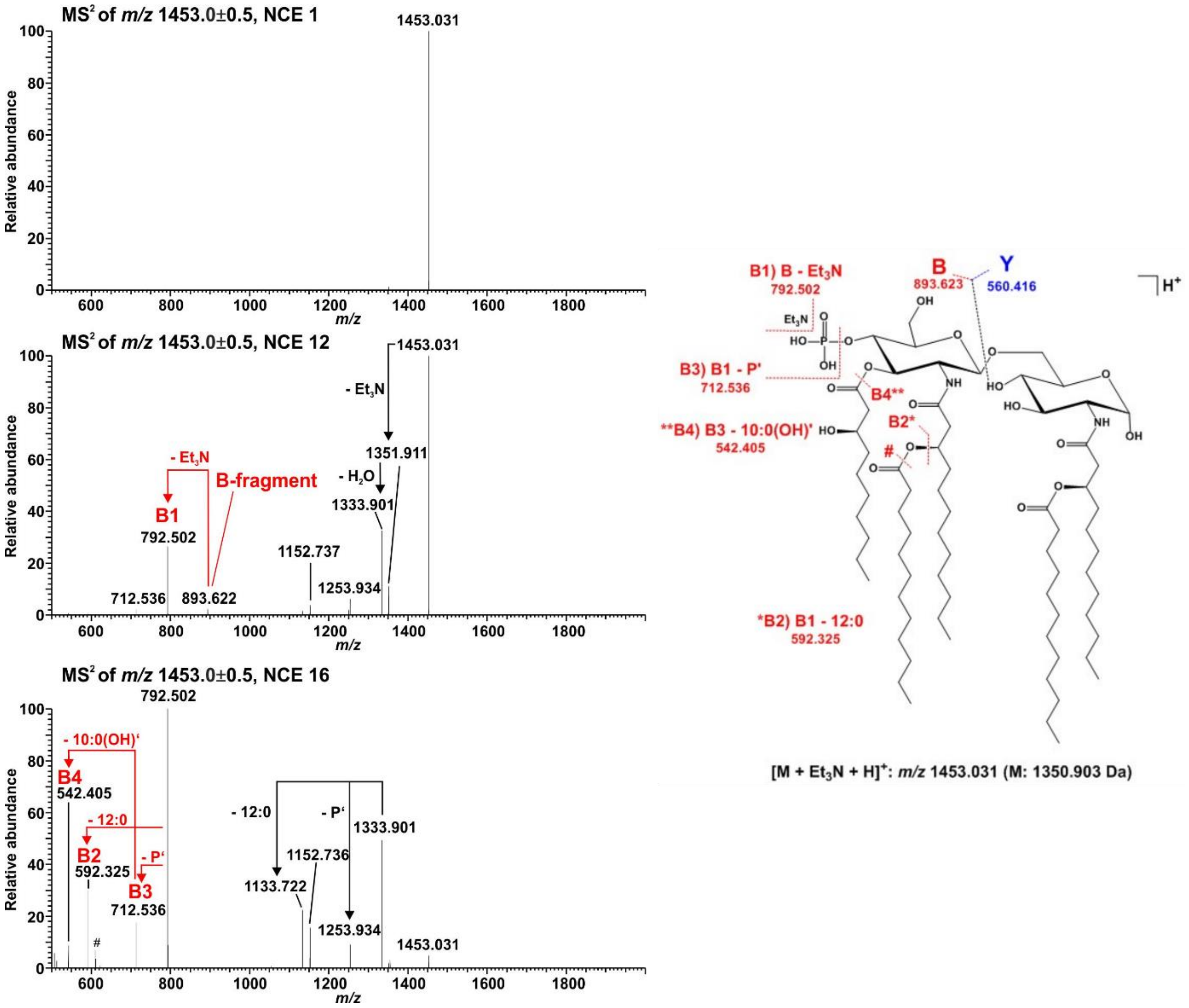

Figure S3. MS² analysis of the penta-acylated, mono-phosphorylated lipid A species with an monoisotopic neutral mass of 1350.903 Da, analogously performed as experiments shown in Figures 5 and 6. Due to the presence of only one phosphate moiety, only a mono-Et_3N adduct is formed, which has been selected for the MS² experiment (*m*/*z* 1453.031; top panel). The same B-fragment (*m*/*z* 893.622 (with Et_3N) and *m*/*z* 792.502 (without Et_3N)) as observed in the fragmentations of the other lipid A species (Figures 5 and 6) can be observed (middle panel). Here, no corresponding Y-fragment ion can be detected, most likely due to the absence of a charge-stabilizing functional group. Nonetheless, the B-fragment and its further fragmentation (bottom panel) is indicative for the fact that the mono-phosphorylated lipid A lacks the 1-*P*.

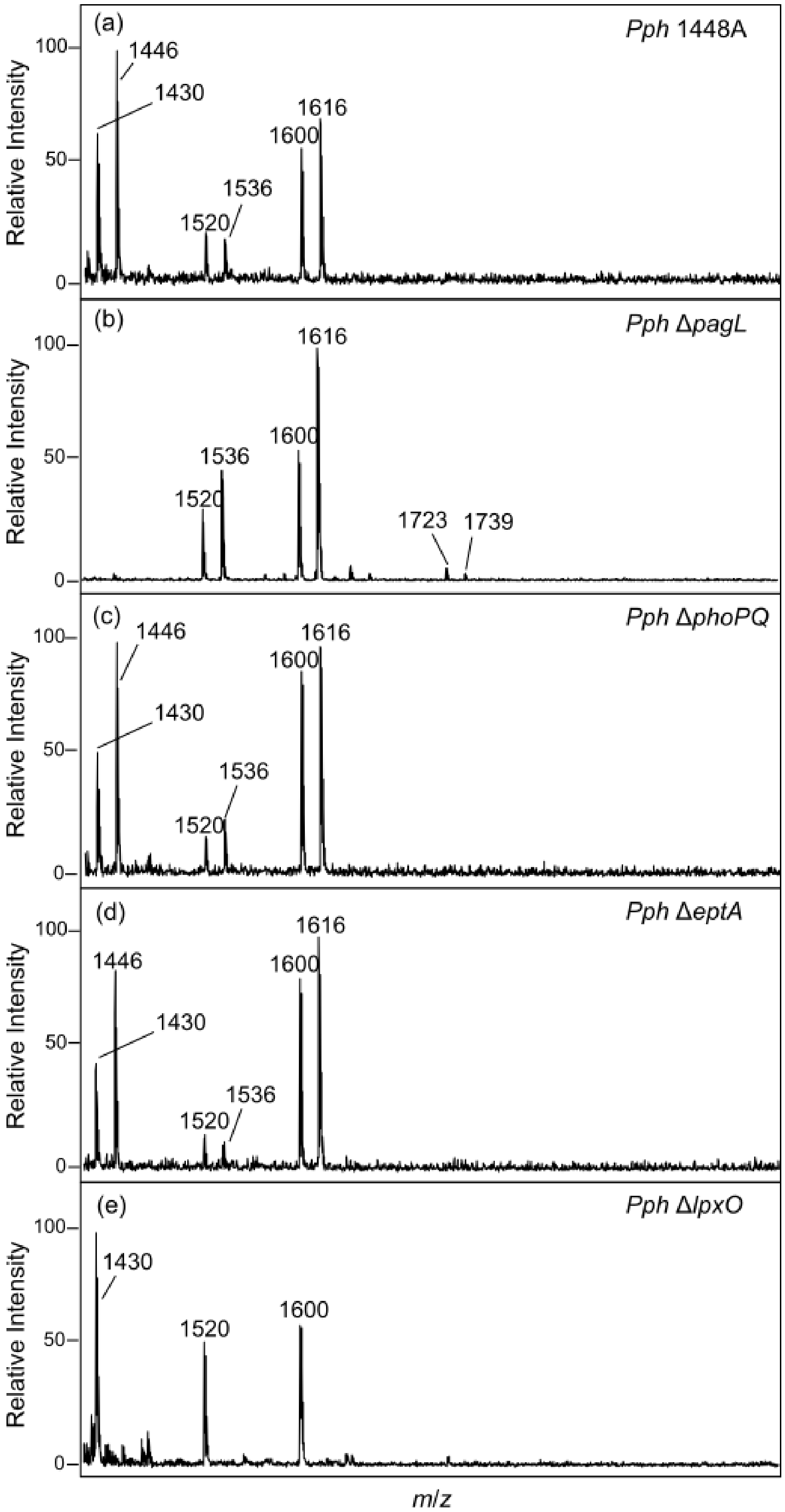

Figure S4. Mass spectrometric analysis of crude lipid A preparations generated from *Pph* 1448A WT and mutant strains via MALDI-TOF. *P*-EtN modifications are underrepresented using this methodology, so only the structural alterations in the Δ*pagL* and Δ*lpxO* mutant can be substantiated.

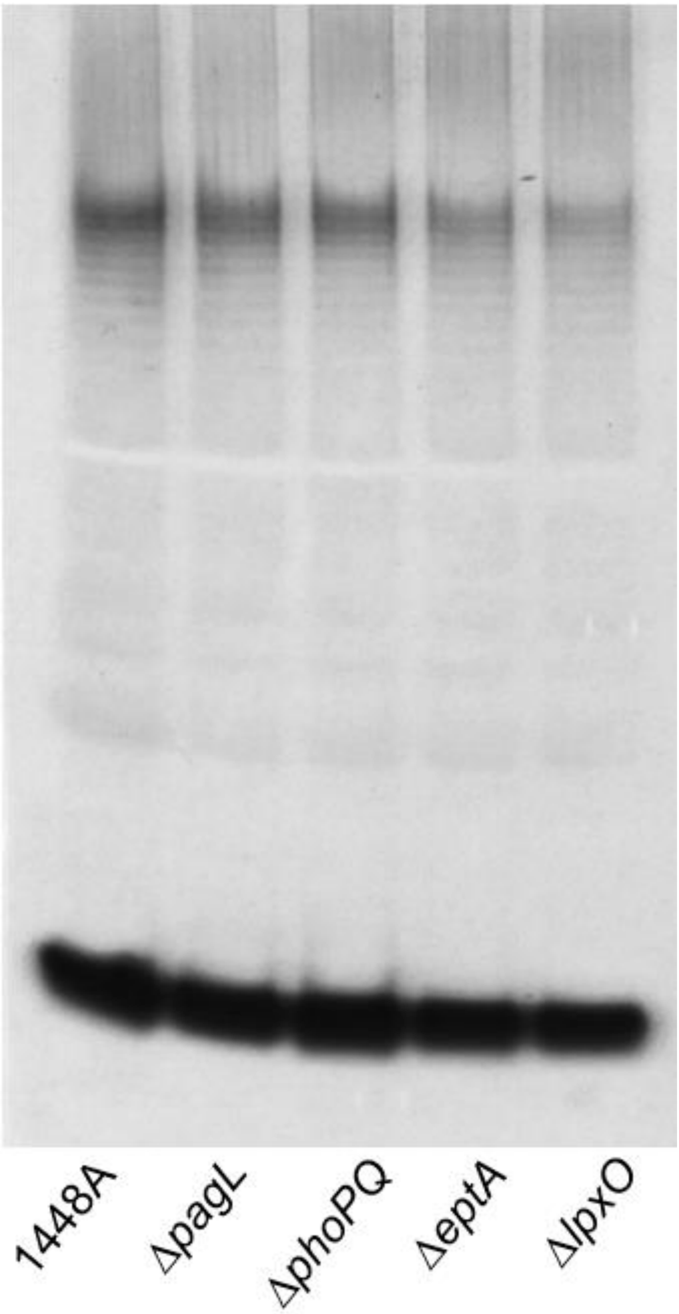

Figure S5. Bis-Tris SDS-PAGE of crude LPS fractions from *Pph* 1448A WT and the indicated mutant strains. OPS with similar banding patterns to the WT could be detected in every strain**.**

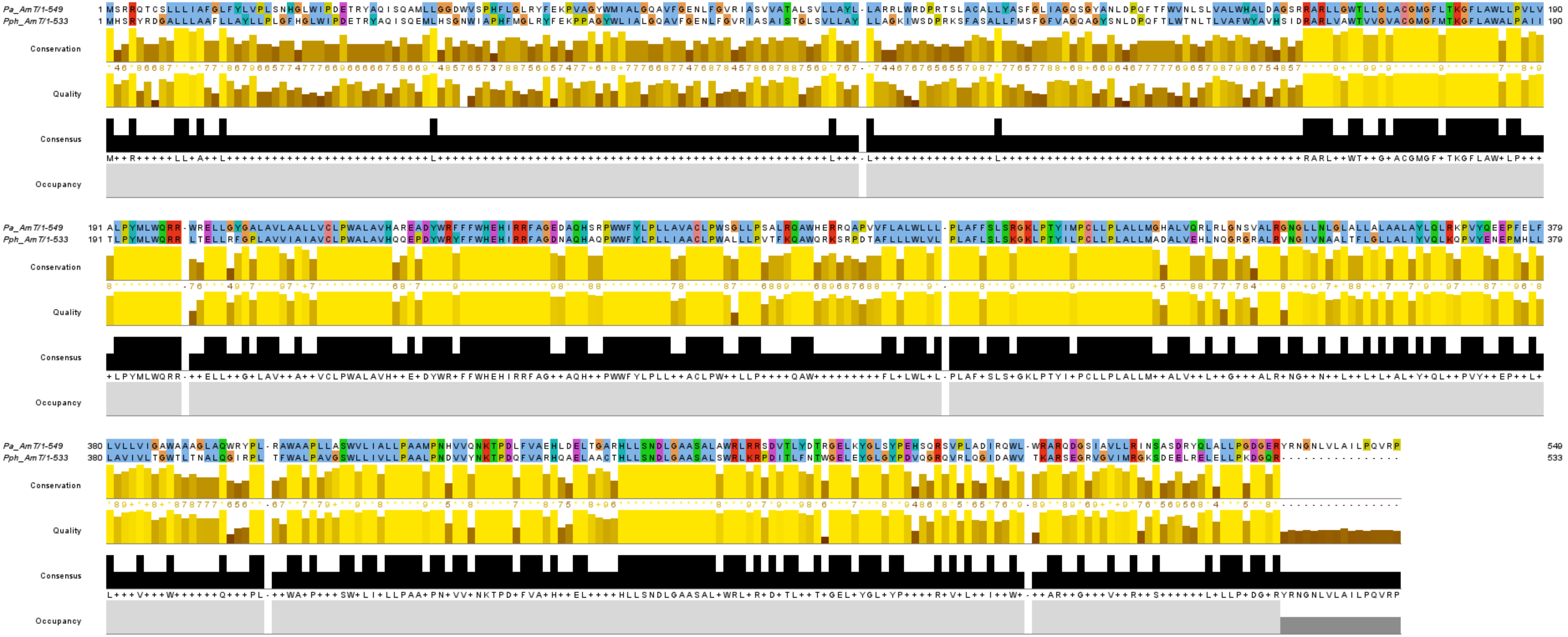

Figure S6. Amino acid sequence alignment of ArnT of *P. aeruginosa* PAO1 and *Pph* 1448A. Sequences were aligned using Jalview with Clustalx as color coding. The sequence of *P. aeruginosa* PAO1 ArnT exceeds *Pph* 1448A ArnT by 16 amino acids.

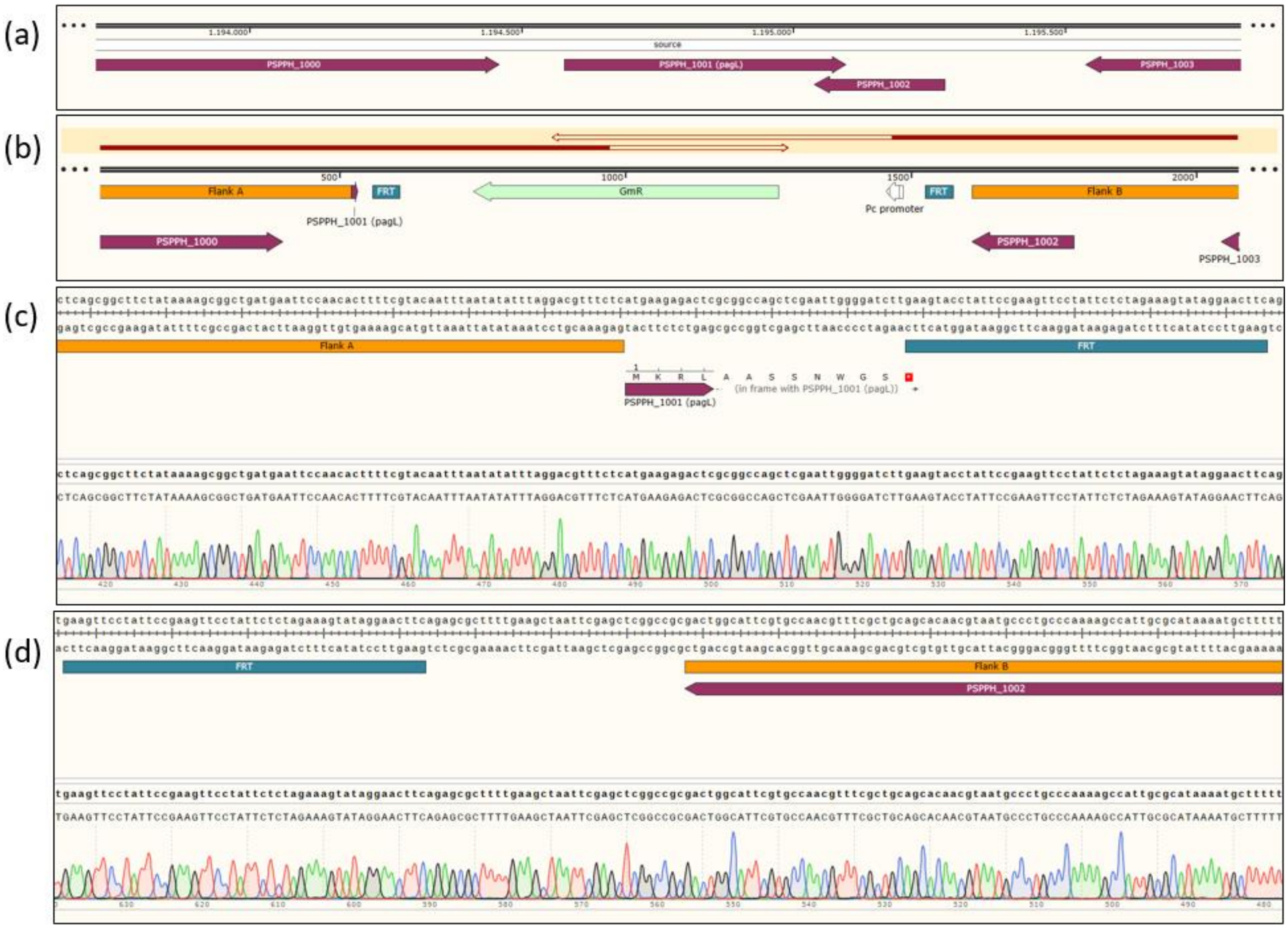

Figure S7. Verification of the *pagL* knockout and gentamicin knock-in in the *Pph* 1448A genome by Sanger sequencing. (**a**) Genetic map showing *Pph* 1448A WT *pagL* (PSPPH_1001) and neighboring genes. Notably, the open reading frames of PSPPH_1001 and PSPPH_1002 overlap. PSPPH_1002 was computationally predicted and no gene function was predicted or shown. (**b**) Modified *Pph* 1448A genome with a gentamicin (Gm^R) knock-in replacing the putative *Pph* 1448A *pagL*. Red arrows indicate sequencing coverage to verify the gentamicin knock-in and the *pagL* knockout, respectively. FRT sequences flanking the gentamicin cassette can be used for site-directed recombination. (**c, d**) Transition region of the flanking regions A (**c**) and B (**d**) to the gentamicin selection marker. Sequencing chromatograms matching the knock-in are shown below the predicted sequence. The residual PagL sequence in combination with the introduced fragment results in a premature stop codon.

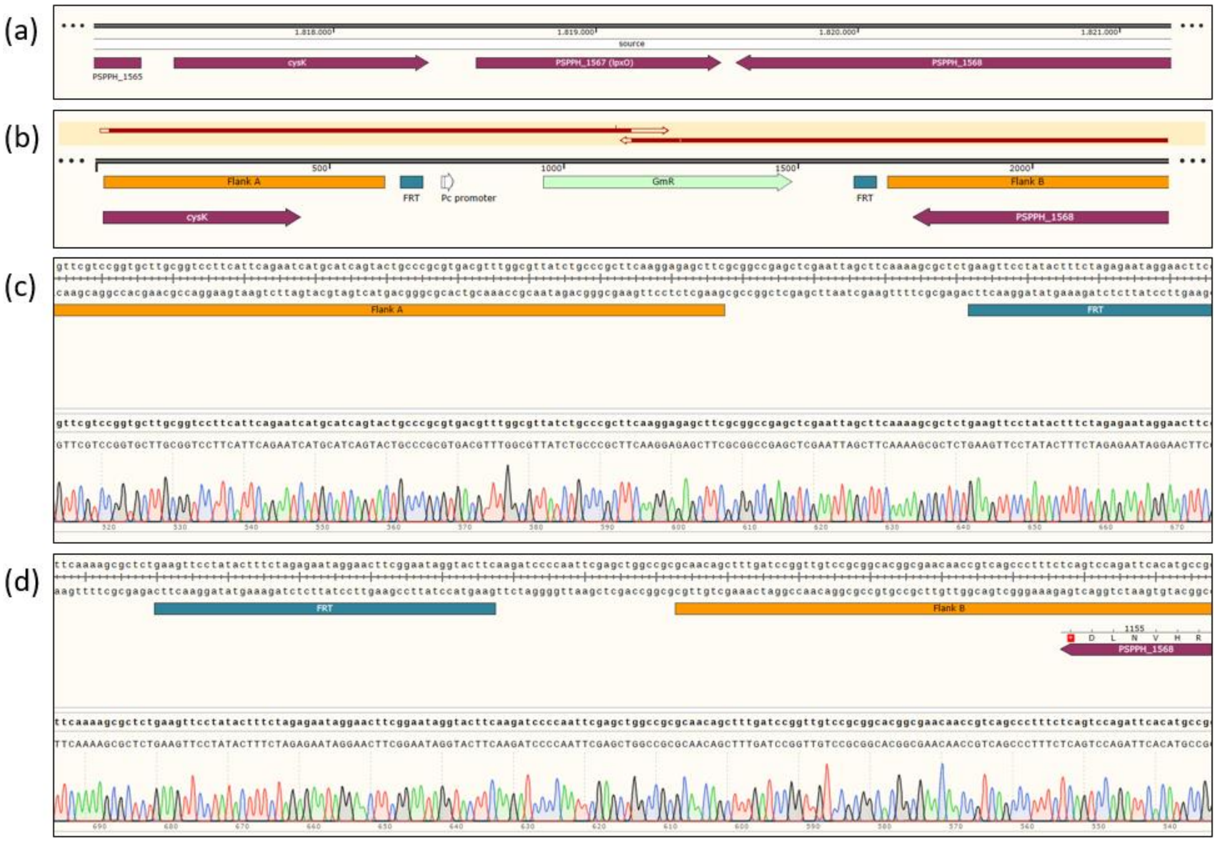

Figure S8. Verification of the *lpxO* knockout and gentamicin knock-in in the *Pph* 1448A genome by Sanger sequencing. (**a**) Genetic map showing *Pph* 1448A WT *lpxO* (PSPPH_1567) and neighboring genes. (**b**) Modified *Pph* 1448A genome with a gentamicin (Gm^R) knock-in replacing the putative *Pph* 1448A *lpxO*. Red arrows indicate sequencing coverage to verify the gentamicin knock-in and the *lpxO* knockout, respectively. FRT sequences flanking the gentamicin cassette can be used for site-directed recombination. (**c, d**) Transition region of the flanking regions A (**c**) and B (**d**) to the gentamicin selection marker. Sequencing chromatograms matching the knock-in are shown below the predicted sequence.

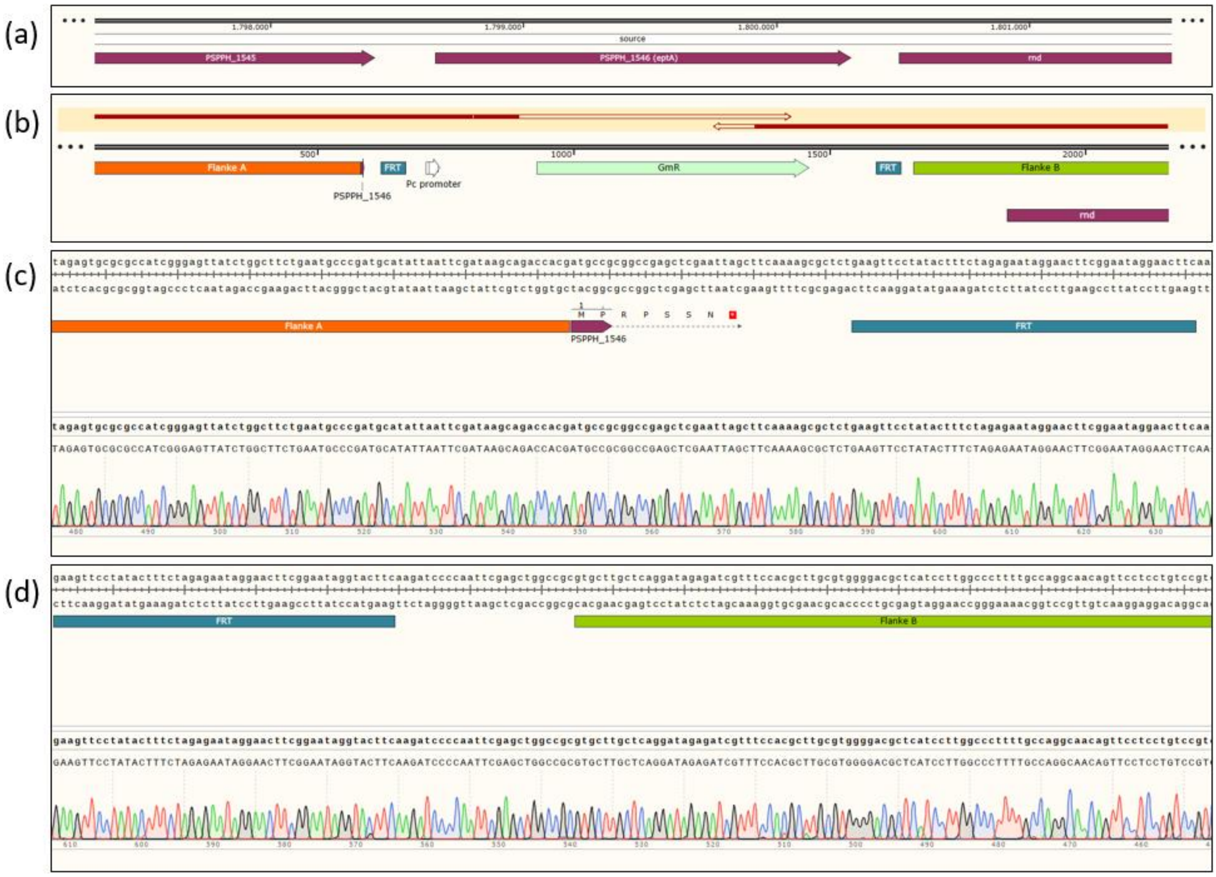

Figure S9. Verification of the *eptA* knockout and gentamicin knock-in in the *Pph* 1448A genome by Sanger sequencing. (**a**) Genetic map showing *Pph* 1448A WT *eptA* (PSPPH_1546) and neighboring genes. (**b**) Modified *Pph* 1448A genome with a gentamicin (Gm^R) knock-in replacing the putative *Pph* 1448A *eptA*. Red arrows indicate sequencing coverage to verify the gentamicin knock-in and the *eptA* knockout, respectively. FRT sequences flanking the gentamicin cassette can be used for site-directed recombination. (**c, d**) Transition region of the flanking regions A (**c**) and B (**d**) to the gentamicin selection marker. Sequencing chromatograms matching the knock-in are shown below the predicted sequence. The residual EptA sequence in combination with the introduced results in a premature stop codon.

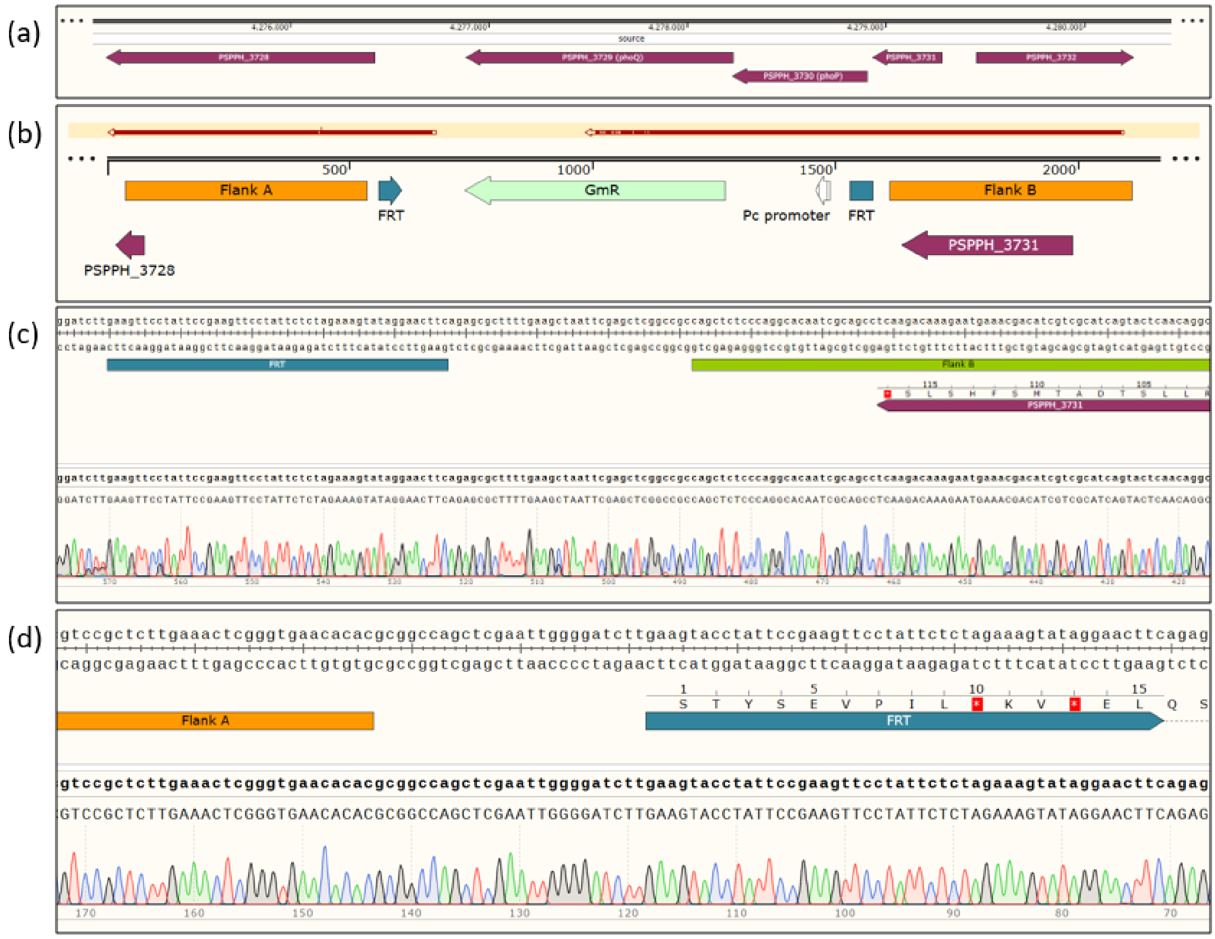

Figure S10. Verification of the *phoP*/*phoQ* knockout and gentamicin knock-in in the *Pph* 1448A genome by Sanger sequencing. (**a**) Genetic map showing *Pph* 1448A WT *phoP* (PSPPH_3730) and *phoQ* (PSPPH_3729), as well as neighboring genes. (**b**) Modified *Pph* 1448A genome with a gentamicin (Gm^R) knock-in replacing the putative *Pph* 1448A *phoP* and *phoQ*. Red arrows indicate sequencing coverage to verify the gentamicin knock-in and the *phoP*/*phoQ* knockout, respectively. FRT sequences flanking the gentamicin cassette can be used for site-directed recombination. (**c, d**) Transition region of the flanking regions A (**c**) and B (**d**) to the gentamicin selection marker. Sequencing chromatograms matching the knock-in are shown below the predicted sequence.

Table S1. Analysis of predicted proteomes for sequence identity (in %) with proteins involved in lipid A biosynthesis, modification, or regulation. *P. aeruginosa* PAO1 was used as reference.

Gene locus (encoded proteine) of *P. aeruginosa* PAO1	*P. syringae* pv. *actinidiae* KW41	*P. syringae* pv. *maculicola* ES4326	*P. syringae* pv. *phaseolicola* 1448A	*P. syringae* pv. *syringae* B728a	*P. syringae* pv. *tomato* DC3000	*P. syringae* pv. *tomato* T1	*P. cichorii* JBC1	*P. fluorescens* A506	*P. fluorescens* FR1	*P. fluorescens* Pf0-1	*P. fuscovaginae* SE-1	*P. putida* KT2440	*P. savastanoi* pv. *savastanoi* NCPPB 3335	*P. viridiflava* UASWS0038	*P. aeruginosa* PA14
PA3644 (LpxA)	88.0	88.4	87.6 (PSPPH_3828)	88.0	87.6	87.6	87.2	90.7	91.5	90.7	90.3	86.4	88.0	87.6	100.0
PA4406 (LpxC)	90.4	90.4	91.1 (PSPPH_4102)	91.1	90.4	90.4	90.1	91.4	91.4	91.7	92.1	91.7	91.1	90.8	99.7
PA3646 (LpxD)	75.6	76.5	74.5 (PSPPH_3830)	75.4	76.2	76.2	79.8	77.7	80.2	79.9	79.4	80.2	75.1	78.4	99.4
PA1792 (LpxH)	74.6	73.7	72.9 (PSPPH_1676)	72.9	74.6	73.3	72.5	73.7	72.9	75.0	76.5	73.3	72.9	72.0	100.0
PA3643 (LpxB)	79.2	77.2	77.3 (PSPPH_3827)	76.9	79.2	79.5	78.1	79.0	76.9	77.4	79.1	80.2	77.1	76.8	99.7
PA2981 (LpxK)	75.4	76.3	77.2 (PSPPH_1628)	76.6	75.4	75.4	75.5	75.2	73.9	74.8	78.4	73.8	77.2	75.1	100.0
PA3242 (LpxL/HtrB2)	78.4	80.0	80.3 (PSPPH_1607)	79.4	78.7	78.7	76.1	80.4	79.9	80.3	79.4	78.8	80.3	78.7	99.4
PA0011 (LpxM/HtrB1)	80.0	79.3	79.0 (PSPPH_0014)	79.0	79.3	79.3	79.0	76.9	76.3	75.6	77.6	73.9	79.0	80.0	100.0
PA4661 (PagL)	60.7	67.7	59.5 (PSPPH_1001)	60.1	64.0	60.7	61.3	61.3	59.5	60.7	60.1	67.6	60.1	61.9	100.0
PA4512 (LpxO1)	55.1	51.7	54.0 (PSPPH_1567)	54.7	55.1	55.1	55.4	71.8	72.9	71.6	71.9	69.4	54.0	55.1	99.3
PA0936 (LpxO2)	73.4	73.4	73.4 (PSPPH_1567)	73.7	73.4	73.4	71.2	75.3	74.3	75.0	75.0	76.5	73.4	73.4	99.7
PA1972 (EptA)	63.6	61.9	63.5 (PSPPH_1546)	63.5	63.5	63.5	63.9	28.1	65.9	63.2	29.3	61.2	63.6	62.7	99.6
PA3556 (ArnT)	63.1	60.6	63.1 (PSPPH_2806)	62.5	25.5	—	—	62.3	27.4	62.2	61.5	26.7	62.1	61.3	99.1
PA1343 (PagP)	—	—	—	—	—	—	—	65.1	—	—	67.8	—	—	—	98.7
PA1179 (PhoP)	83.5	83.0	83.5 (PSPPH_3730)	83.5	83.5	83.5	85.7	84.4	85.8	84.8	83.6	86.7	83.5	84.4	100.0
PA1180 (PhoQ)	65.5	67.9	65.2 (PSPPH_3729)	65.2	65.4	65.4	65.6	67.2	68.1	66.7	67.3	67.4	64.9	64.3	100.0
PA4776 (PmrA)	57.5	57.9	57.5 (PSPPH_3453)	57.9	57.0	57.5	55.6	54.2	55.5	55.5	55.1	56.8	57.5	53.2	100.0
PA4777 (PmrB)	30.9	32.8	31.8 (PSPPH_3454)	31.1	31.5	31.5	32.0	37.8	27.2	27.6	37.8	35.8	31.6	32.4	99.0

Table S2. Strains used in this study.

Strain	Abbreviation	Source / reference
Pseudomonas syringae		
Pph 1448A (race 6)	WT or 1448A	[57]
Pph pagL::Gm^R	Δ*pagL*	generated in this study
Pph phoPQ::Gm^R	Δ*phoPQ*	generated in this study
Pph lpxO::Gm^R	Δ*lpxO*	generated in this study
Pph eptA::Gm^R	Δ*eptA*	generated in this study
Escherichia coli		
E. coli DH5α	DH5α	[61]
E. coli HB101	HB101	[63]

Table S3. Oligonucleotides used in this study.

Purpose	Name	Sequence (5′ → 3′)
Sequencing of pGGKO plasmid	seqPGGKO-f	agcgcccaatacgcaaac
	seqPGGKO-r	cagtagctgacattcatc
GmR insertion into pGGKO	ggGmR-f	tgaagacttggccagctcgaattggggatcttg
	ggGmR-r	tgaagacttggccgagctcgaattagcttcaaaagc
Knockout (KO) verification	Vf_flA	gttaccaccgctgcgttcggtc
	Vf_flB	gttaccaccgctgcgttcggtc
pagL KO flanking sequences; cloning primer for insertion into pGGKO	pph-pagL-flA-f	ttgaagactgtcgagttcgggtagtgacccag
	pph-pagL-flA-r	ttgaagacgcggccgcgagtctcttcatgagaaacgtcc
	pph-pagL-flB-f	ttgaagacgcggccgcgactggcattcgtgccaac
	pph-pagL-flB-r	ttgaagacgtctaggaacgcaaagggattgcgac
pagL KO verification	vf-pph-pagL-f	gttgctggaccgatttgcc
	vf-pph-pagL-r	cgctgatctgccgtgagttc
lpxO KO flanking sequences; cloning primer for insertion into pGGKO	pph-lpxO-flA-f	ttgaagactgtcgaccggtatttcccgctatatcaag
	pph-lpxO-flA-r	ttgaagacgcggccgcgaagctctccttgaagcggg
	pph-lpxO-flB-f	tttgaagacgcggccgcgcaacagctttgatccggttg
	pph-lpxO-flB-r	tttgaagacgtctaggactcacgcagcgaaggg
lpxO KO verification	vf-pph-lpxO-f	gtactggtggcaggcgtcg
	vf-pph-lpxO-r	caaacgcctcgacagccac
phoPQ KO flanking sequences; cloning primer for insertion into pGGKO	pph-phoPQ-flA-f	ttgaagactgtcgagacaatcgcaacgattacctg
	pph-phoPQ-flA-r	ttgaagacgcggccgcgtgtgttcacccgagtttcaag
	pph-phoPQ-flB-f	ttgaagacgcggccgccagctctcccaggcacaatcg
	pph-phoPQ-flB-r	ttgaagacgtctaggctgcatccggtgtagatgac
phoPQ KO verification	Vf_GmR_rv	ggccgcggagttgttcgg
	vf-pph-phoPQ-rv	cgcaaacaaccatagccgattc
eptA KO flanking sequences; cloning primer for insertion into pGGKO	pph-eptA-flA-f	tttgaagactgtcgagcacatcgctcagtgcaac
	pph-eptA-flA-r	tttgaagacgcggccgcggcatcgtggtctgcttatcg
	pph-eptA-flB-f	tttgaagacgcggccgcgtgcttgctcaggatagagatcg
	pph-eptA-flB-r	tttgaagacgtctagcagacgcgaatagcccatgg
eptA KO verification	vf-pph-eptA-fw	cgccggtggagatcaacgg
	vf-pph-eptA-rv	gtcgatgttgaggacctcctgc
